# Peroxisome Proliferator-Activated Receptor-δ Deficiency in Microglia Results in Exacerbated Axonal Injury and Tissue Loss in Experimental Autoimmune Encephalomyelitis

**DOI:** 10.3389/fimmu.2021.570425

**Published:** 2021-02-26

**Authors:** Ellinore R. Doroshenko, Paulina C. Drohomyrecky, Annette Gower, Heather Whetstone, Lindsay S. Cahill, Milan Ganguly, Shoshana Spring, Tae Joon Yi, John G. Sled, Shannon E. Dunn

**Affiliations:** ^1^Department of Immunology, University of Toronto, Toronto, ON, Canada; ^2^Keenan Research Centre for Biomedical Science of St. Michael's Hospital, Toronto, ON, Canada; ^3^Peter Gilgan Centre for Research and Learning, Hospital for Sick Children, Toronto, ON, Canada; ^4^Mouse Imaging Centre, The Hospital for Sick Children, Toronto, ON, Canada; ^5^Histology Core, The Centre for Phenogenomics, Toronto, ON, Canada; ^6^Department of Medical Biophysics, University of Toronto, Toronto, ON, Canada; ^7^Women's College Research Institute, Women's College Hospital, Toronto, ON, Canada

**Keywords:** microglia, experimental autoimmune encephalomyelitis, axon injury, neuroinflammation, peroxisome proliferator-activated receptor

## Abstract

Peroxisome proliferator-activated receptor (PPAR)-δ is a nuclear receptor that functions to maintain metabolic homeostasis, regulate cell growth, and limit the development of excessive inflammation during immune responses. Previously, we reported that PPAR-δ-deficient mice develop a more severe clinical course of experimental autoimmune encephalomyelitis (EAE); however, it was difficult to delineate the role that microglia played in this disease phenotype since PPAR-δ-deficient mice exhibited a number of immune defects that enhanced CNS inflammation upstream of microglia activation. Here, we specifically investigated the role of PPAR-δ in microglia during EAE by using mice where excision of a floxed *Ppard* allele was driven by expression of a tamoxifen (TAM)-inducible CX3C chemokine receptor 1 promoter-Cre recombinase transgene (*Cx3cr1*^CreERT2^: *Ppard*^fl/fl^). We observed that by 30 days of TAM treatment, *Cx3cr1*^CreERT2^: *Ppard*^fl/fl^ mice exhibited Cre-mediated deletion primarily in microglia and this was accompanied by efficient knockdown of *Ppard* expression in these cells. Upon induction of EAE, TAM-treated *Cx3cr1*^CreERT2^: *Ppard*^fl/fl^ mice presented with an exacerbated course of disease compared to TAM-treated *Ppard*^fl/fl^ controls. Histopathological and magnetic resonance (MR) studies on the spinal cord and brains of EAE mice revealed increased Iba-1 immunoreactivity, axonal injury and CNS tissue loss in the TAM-treated *Cx3cr1*^CreERT2^: *Ppard*^fl/fl^ group compared to controls. In early EAE, a time when clinical scores and the infiltration of CD45^+^ leukocytes was equivalent between *Cx3cr1*^CreERT2^: *Ppard*^fl/fl^ and *Ppard*^fl/fl^ mice, *Ppard*-deficient microglia exhibited a more reactive phenotype as evidenced by a shorter maximum process length and lower expression of genes associated with a homeostatic microglia gene signature. In addition, *Ppard*-deficient microglia exhibited increased expression of genes associated with reactive oxygen species generation, phagocytosis and lipid clearance, M2-activation, and promotion of inflammation. Our results therefore suggest that PPAR-δ has an important role in microglia in limiting bystander tissue damage during neuroinflammation.

## Introduction

Multiple sclerosis (MS) is a chronic, autoimmune-based neurodegenerative disease that affects more than 2.5 million people worldwide (1). The majority of MS patients initially present with a relapsing-remitting form of this disease (RRMS) where acute inflammatory episodes are interspersed with periods of disease remission (1). These acute episodes of inflammation correlate with the invasion of T cells and monocytes into the submeningeal and perivascular spaces in the CNS white matter, which is associated with microglia activation, activation of blood brain barrier endothelium, demyelination, and axonal injury (1, 2). With time, the neuronal injury elicited by repeated autoimmune attacks and the gradual seeding of immune cells under the meninges triggers chronic microglia activation and neuronal loss resulting in CNS atrophy and disability progression (3, 4).

Experimental autoimmune encephalomyelitis (EAE) is the most common animal model of MS (5). EAE has been instrumental in modeling the sequence of autoimmune events that lead to myelin and neuronal damage in MS (5). Studies in EAE have also revealed the many roles of microglia in CNS inflammatory and repair processes in this disease (6). While microglia are not considered to be efficient at antigen presentation (7), these cells are crucial to the onset of EAE, since ablation of microglia prevents disease development (8). In EAE, microglia sense immune cell infiltration and damage in the CNS via expression of cytokine receptors and purinergic receptors and, in response to these cues, adopt a more active phenotype (9). As part of this activation process, microglia retract their processes, proliferate (10), and upregulate the phagocytic and antigen presenting machinery and expression of inducible nitric oxide synthase (iNOS) and proinflammatory mediators (6, 11). In addition to these pro-inflammatory activities, microglia are also crucial for healing within lesions via the phagocytic clearance of myelin debris (12). This process of phagocytosis is considered to be a “double-edged sword,” since it is accompanied by an oxidative burst and production of reactive oxygen species (ROS) that can be damaging to myelin and axons (12). Microglia and macrophage production of ROS and reactive nitrogen species are major contributors to neurodegeneration in EAE and MS (13, 14).

PPAR-δ is a member of the PPAR family of nuclear receptors. This transcriptional regulator is ubiquitously expressed and plays diverse roles in regulating cellular metabolism, proliferation and inflammation in the host (15). Previously, it was reported that whole-body deficiency of PPAR-δ in mice resulted in the development of a more progressive form of EAE (16, 17). However, it was difficult to discern how much microglia contributed to this phenotype, since PPAR-δ deficient mice exhibited a number of immune defects that operate upstream of microglia activation in EAE, including the enhanced production of IL-12p40 and IL-6 by spleen macrophages (16), increased lymphopenia and associated memory T cell formation (18), enhanced dendritic cell priming of T helper (Th) cells (19), and heightened IFN-γ and IL-17 production by Th cells (16, 17).

Here, we specifically assessed the role of PPAR-δ in microglia in EAE by generating mice where excision of a floxed *Ppard* allele was driven by a TAM-inducible *Cx3cr1*^CreERT2^ promoter (*Cx3cr1*^CreERT2^*:Ppard*^fl/fl^). We induced EAE in *Cx3cr1*^CreERT2^*:Ppard*^fl/fl^ and *Ppard*^fl/fl^ mice at 30 days following TAM treatment, a time when the expression of the Cre transgene was most prominent in microglia. We observed that *Cx3cr1*^CreERT2^*:Ppard*^fl/fl^ mice developed a similar onset of EAE as compared to *Ppard*^fl/fl^ controls; however, in the post-acute phase of disease, the clinical scores remained elevated in the male *Cx3cr1*^CreERT2^:*Ppard*^fl/fl^ mice, while male *Ppard*^fl/fl^ controls regained hindlimb function. Histological analysis of the spinal cord showed that *Cx3cr1*^CreERT2^:*Ppard*^fl/fl^ mice, regardless of sex, exhibited enhanced myelin and axon injury compared to *Ppard*^fl/fl^ counterparts. *In situ* analysis coupled with flow cytometry, and gene profiling studies showed that PPAR-δ-deficiency in microglia was associated with a shift away from a homeostatic microglia phenotype and heightened expression of genes associated with ROS generation, phagocytosis and lipid clearance, M2-activation, and promotion of inflammation. Together, these results demonstrate that PPAR-δ operates in microglia to limit the development of excessive CNS tissue damage during neuroinflammation.

## Materials and Methods

### Mice and Ethics

Mice that have a targeted neomycin disruption of the final exon of the PPAR-δ gene (*Ppard*^−/−^) have been described previously (20). These mice express a truncated unstable *Ppard* transcript and undetectable PPAR-δ protein levels. Homozygous mutant mice on a mixed SV.129/C57BL/6 background (estimated two generations to C57BL/6) (16) were crossed an additional 6 generations to C57BL/6. Heterozygotes were used to develop the homozygous *Ppard*^−/−^ and *Ppard*^+/+^ mice used for microglial cultures. Mice with a microglia-enriched deletion of PPAR-δ were generated by crossing mice that express a floxed exon 4 of the PPAR-δ gene (21) (*Ppard*^fl/fl^; Jackson Laboratory) with transgenic mice carrying a tamoxifen-inducible Cre-ERT2 fusion protein and a constitutively-expressed enhanced yellow fluorescent protein (EYFP) driven by the endogenous *Cx3cr1* promoter (*Cx3cr1*^CreERT2^, Jackson Laboratory) (22). Mice heterozygote for *Cx3cr1*^CreERT2^ and homozygous for the floxed PPAR-δ allele (*Cx3cr1*^CreERT2^:*Ppard*^fl/fl^) were identified in the F2 generation and were crossed with homozygote *Ppard*^fl/fl^ mice to generate littermate *Cx3cr1*^CreERT2^:*Ppard*^fl/fl^ and *Ppard*^fl/fl^ mice for use in experiments. *Cx3cr1*^CreERT2^ mice were also crossed to mice expressing td-Tomato preceded by a floxed stop codon under the control of the Rosa26 locus (*R26-td-Tomato*, #07909) (23) to evaluate recombination efficiency in microglia and other myeloid cell populations. PCR genotyping of tail DNA was performed for *Ppard*^−/−^ mice as previously described (16) and for all other mice using the Jackson Laboratory master protocols. Mice were housed within a specific pathogen-free facility at the University Health Network and St. Michael's Hospital. Experiments were performed under animal use protocols (AUPs 2863, 5937 at UHN or #994 at St. Michael's hospital) that were approved by site-specific animal ethics committees.

### Isolation and Culture of Primary Microglia

Microglia were isolated from the brain and spinal cords of 5–7 weeks old mice according Ponomarev et al. (24) with the following modifications. Instead of homogenization, CNS tissue was scissor-minced in 1 × HBSS (1.5 mL/brain and spinal cord) and digested with *Clostridium histolyticum* collagenase type IV (300 U/mL final) + 5 U/ml DNAse (Sigma) for 30 min prior to filtration through a 70 micron sieve. Isolated microglia cells were plated in 12-well plates (0.25 × 10^6^ cells per well) in complete DMEM (Cellgro) (containing 10% FCS, 2 mM glutamine, 50 μM 2-mercaptoethanol, 50 μg/ml gentamicin, all from Life Technologies) that also contained 10 ng/ml macrophage colony-stimulating factor (M-CSF) (R&D Systems). Microglia were expanded as described (24), with the exception that at the time of the first passage, cells were detached from the plate using a cell scraper and were purified using EasySep™ Mouse CD11b Positive Selection Kit (STEMCELL Technologies) prior to further expansion in 12-well plates (0.1 × 10^6^/well) in complete DMEM containing M-CSF (10 ng/ml). Cultured microglia were used for experimental studies after 2 or 3 passages.

### *In vitro* BrdU Incorporation and CFSE Dilution Assays

For BrdU incorporation assays, cultured microglia were resuspended in complete DMEM containing 10 ng/ml M-CSF and were cultured (0.1 × 10^6^/well) in 24 well plates with 0.1 mM BrdU for 24 or 48 h. For CFSE dilution assays, microglia were labeled with 0.434 μM CFSE in 37°C 1 x PBS for 20 min (Thermofisher) according to the product directions and were cultured in complete DMEM containing 10 ng/ml M-CSF at 37°C for 24, 48, and 72 h. At the appropriate time point, cells were detached from plates, were washed with FACS buffer (1 × PBS with 2% FCS) and then stained and analyzed by flow cytometry as follows.

All staining steps were performed at 4°C in the dark, centrifugation steps were all at 524 × g or 5 min at 4°C, and staining volumes were 100 μl. Cells (1 × 10^6^/stain) were first blocked with anti-CD16/CD32 (5 μg/ml) (ThermoFisher) in FACS buffer. Cells were washed in 200 μl FACS buffer and then stained for 30 min in FACS buffer containing anti-mouse CD11b (M1/70, ThermoFisher), CD45 (30-F11 ThermoFisher) and Fixable Viability Dye eFluor506 (diluted 1:1,000, ThermoFisher). Intranuclear staining for BrdU was measured using the APC BrdU kit (BD Bioscience). Cells were washed twice with FACS buffer prior to flow cytometry acquisition. Data were analyzed using Flowjo (Flowjo LLC).

### Microglia Stimulation *in vitro*

For microglia activation studies, cells were harvested from plates and were resuspended in complete DMEM without M-CSF, and then plated in triplicate on to 96-well flat bottom plates at a density of 25,000 cells/well in the presence of the Th1 cytokine IFN-γ (150 U/mL) (Thermofisher). The following day, 10 ng/ml LPS (Sigma) or equivolume media was added to the cultures. Supernatants were harvested at 6 h or 24 h. ELISA kits were used to measure prostaglandin E2 (Cayman Chemical, Cat#500141) and CXCL10 (Thermofisher, Cat # BMS6018), RANTES (ThermoFisher, Cat#KMC1031), and cytokines (Ready-Set-Go ELISA kits, Thermofisher) in culture supernatants. Nitrite levels were assessed by Griess assay on 24 h supernatants (25) with absorbance of the dye measured at 560 nm. To ensure that equivalent cell numbers were plated, at the end of the experiment, supernatants were removed and replaced with 50 μl of crystal violet solution (0.5% crystal violet, in methanol) (Sigma) for 15 min at room temperature. Cells were then washed with tap water, the incorporated dye solubilized with sodium citrate solution (0.1 M in 50% ethanol), and the absorbance was read at 560 nm.

### Tamoxifen Treatment

TAM (Sigma) was solubilized in ethanol and diluted ten-fold in corn oil for a final concentration of 100 mg/mL and was heated to 55°C and agitated periodically until the powder was completely dissolved. Mice (6 weeks of age) were gavaged twice with 100 μl using a ball-tipped 1.5 inch 20 G feeding needle, with each dose spaced by 2 days. Experiments were initiated after a minimum of 30 days post-TAM treatment.

### FACS Sort of Microglia for Analysis of Gene Expression

For real-time analysis of *Ppard* expression in microglia, total CNS mononuclear cells were isolated from the brains and spinal cords of TAM-treated *Cx3cr1*^CreERT2^:*Ppard*^fl/fl^ and *Ppard*^fl/fl^ mice by collagenase digestion followed by centrifugation through a discontinuous Percoll gradient (16). Cells were stained with CD11b and CD45 antibodies and DAPI and CD11b^+^CD45^lo^DAPI^−^ cells were sorted to purity using a MoFlo XDP Cell sorter (Beckman Coulter). For real-time PCR, RNA was isolated using an RNeasy Miniprep Kit (Qiagen). Total RNA (100 μg) was reverse transcribed to cDNA using SuperScript III reverse transcriptase (Invitrogen) and cDNA abundance was analyzed by real-time PCR using Roche SYBR Green I master mix and sequence specific primers as described previously (19).

For RNA sequencing studies, *N* = 9 TAM-treated male *Cx3cr1*^CreERT2^:*Ppard*^fl/fl^ and *Ppard*^fl/fl^ mice per group were euthanized at 2–4 days post-onset of disease. Mice were matched for disease duration and clinical scores. *N* = 3 spinal cords were pooled together per sample and CNS mononuclear cells were isolated and microglia were FACS sorted from mononuclear cells as described. RNA was isolated from sorted microglia using the PicoPure™ RNA Isolation Kit (Thermo Fisher Scientific). RNA quality was evaluated using the 2100 Bioanalyzer (Agilent) and samples with RIN numbers > 7 were submitted for sequencing and analysis at the UHN Bioinformatics and HPC Core, Princess Margaret Cancer Center. Samples were analyzed using the Tuxedo package (26). In brief, samples were aligned to the mouse reference genome GRCm38 using HISAT2, transcript assembly was performed with Stringtie, and differential gene expression analysis was performed with ballgown. Raw and processed data were deposited in Geo (accession number GSE164702). A two-tailed Mann-Whitney test was also performed to expand the list of differentially-expressed genes (DEGs) to support functional annotation analysis in DAVID (https://david.ncifcrf.gov).

### EAE Induction and Clinical Scoring

EAE was induced after a minimum of 30 days post TAM administration by subcutaneous immunization at two sites of the chest with an emulsion that contained 100 μg MOG p35-55 (Genemed Synthesis) and Complete Freund's Adjuvant containing 200 μg of heat-killed *Mycobacterium tuberculosis* (H37RA, Difco Laboratories). Mice were also injected with *Bordetella pertussis* toxin (PTX) (List Biologicals, dose used was lot-dependent, but varied between 100 and 250 ng) on days 0 and 2 following immunization. Mice were examined daily for clinical signs of EAE using a five point scale: (0) no clinical signs; (1) limp tail; (2) hindlimb or foot weakness; (3) complete hindlimb paralysis in one or both hindlimbs; (4) hindlimb paralysis plus some forelimb weakness; (5) moribund or dead. Mice that died from EAE were assigned a score of five daily until the end-point of the experiment.

### Measuring Myelin-Specific Responses in T Cells During EAE

Spleens were isolated from mice at 9 days post-immunization with MOG p35-55/CFA and were dissociated into a single cell suspension as described previously (16). Cells were resuspended in complete RPMI, were counted, and plated in 96 well plates (0.5 × 10^6^/well) together with 0, 2, 5, and 10 μg/ml MOG p35-55. Cytokines were measured in culture supernatants at optimal time points (IFN-γ at 48 h, IL-17A at 72 h) using Ready-Set-Go ELISA kits (ThermoFisher). Proliferation was measured using a [^3^H]-thymidine incorporation assay as described previously (16). Cells were pulsed at 48 h of culture and were harvested 18 h later for measurement of radioactivity in counts per min using a beta counter.

### Hematoxylin and Luxol Fast Blue Staining, Immunohistochemistry (IHC), and Immunofluorescence (IF)

Brains and spinal cords were isolated from mice, preserved in 10% neutral buffered formalin (Sigma), and processed in paraffin, and embedded in a single paraffin block (10–12 spinal cord and 6 brain sections/block). Cross-sections (5 μm) were cut (Center for Phenogenomics, Toronto, ON) and dually stained with hematoxylin and eosin (H&E) and Luxol Fast Blue (LFB) to visualize inflammatory/demyelinating lesions. Inflammation and demyelination were scored as described previously (19). IHC for mouse SMI-31 (Biolegend, Cat# 801601, 1:5,000) and SMI-32 (Biolegend; Cat #801701, 1:5,000) antibodies was performed as previously described using the mouse-on-mouse (M.O.M.) kit (Vectorlabs) (27). After incubation with secondary antibodies, sections were probed with streptavidin-HRP (ABC kit, Vectorlabs), developed with DAB (Vectorlabs), and counterstained with Meyer's hematoxylin. Sections were then dehydrated in successive ethanol baths, cleared in xylene, and mounted with Permount.

IF of microglia was performed as follows. Cross-sections of the thoracic spinal cord were de-waxed and subjected to heat-activated, citrate antigen-retrieval (10 mM trisodium citrate, 0.05% Tween 20, pH = 6), were washed in TBS and blocked in 10% Goat serum for 20 min at room temperature prior to incubation overnight at 4°C with rabbit anti-Iba-1 alone (1:8,000, ab178847, Abcam) or with a cocktail of mouse anti-Iba-1 (1:750, EMD Millipore, Cat # MABN92) and mouse anti-TMEM119 (1:100, Abcam, Cat # ab209064). The following day, sections were washed and probed with either goat, anti-rabbit Alexa 568 (1:1,000, Thermofisher) (in the case of rabbit anti-Iba-1), or a secondary antibody cocktail of Goat anti-Rabbit Alexa Fluor 488 (Cat # A11008, Thermo Fisher Scientific) & Goat anti-Alexa Fluor 555 (Cat # ab150118, Abcam) for dual detection of TMEM and Iba-1. Nuclei were stained with DAPI and slides were cover slipped using Vectashield Vibrance mounting medium (Vector Laboratories). Images were captured at 20 or 40 × using a Zeiss slidescanner (AxioScan.Z1).

### Scoring of Inflammation, Demyelination, Axonal Injury and Loss

For measurement of axon injury, spinal cord cross-sections were imaged at 40 × using a microscope (model BX50) and camera (model, DP72, Olympus) and cellSens software (Standard 1.14, Olympus). Images were then opened in Fiji. All SMI-32^+^ ovoids detected in spinal cord section white matter (10–12/mouse) were counted using the count tool and this number was divided by the area of white matter area that was traced using the polygon tool in Fiji (27). Axon counting was done at the level of the thoracic spinal cord in male mice only. For this analysis, four images were captured (60 ×) in the anterior, posterior, and two sides of each spinal cord cross-section (2–3 sections per mouse). Images were opened in Fiji and individual SMI-31^+^ axons were manually counted in a 20,000 μm^2^ frame placed in the center each image. The number of axons was expressed per area of white matter sampled. Histological analyses were performed by an experimenter who was blinded to the identity of each treatment group.

### *Ex vivo* MR Imaging of Mouse Brain and Spinal Cord Specimens

Mice were anesthetized using isoflurane and were transcardially perfused with 4% paraformaldehyde (PFA) fixative containing 2 mM Prohance (Bracco Diagnostics, Inc., NJ, USA) as described previously (28). Mice were decapitated and spinal cord and brains were prepared for MR (28). T2-weighted images were acquired using multi-channel 7.0 T, 40 cm diameter bore magnet (Varian Inc., Palo Alto, CA) with a custom-built 16-coil solenoid array was (29) (MICe, Hospital for Sick Children). The imaging protocol consisted of a 3D fast-spin echo sequence using a cylindrical *k*-space acquisition (30) with the following parameters: repetition time = 350 ms, echo time = 12 ms, echo train length = 6, four averages, field-of-view = 2.0 cm × 2.0 cm × 2.5 cm, matrix size = 504 × 504 × 630, isotropic image resolution = 40 μm.

For the brain images, an automated image registration approach using the advanced normalization tools deformation algorithm (31) was used to assess anatomical differences between groups (32). The images were iteratively registered together to produce a consensus average image of all of the scans. The registration yielded deformation fields for each individual brain, and the Jacobian determinants of these deformation fields provided an estimate of the local volume expansion/contraction at every voxel. In addition, a segmented anatomical atlas with 62 labeled structures (33) was registered to the average image using multiple templates of this segmented atlas (the MAGeT procedure) (34) and from the final voted segmentation, volume changes were calculated and expressed as absolute volumes (mm^3^). In addition, brain volumes were normalized to total brain volume and these relative volumes were compared between groups. Multiple comparisons were controlled for using the false discovery rate (FDR) (35) and statistical significance was defined at an FDR threshold of 10%. The scanned spinal cord images were examined using OCCIviewer (Sunnybrook Research Institute) to determine the anatomical location of the slices. The upper thoracic spinal cord (T1-T7) was maintained throughout all scans and spinal cord morphology was determined using the Allen Brain Atlas Data Portal. Next, the total spinal cord volume, total gray matter volume, and total white matter volume of the thoracic spinal cord region of interest for all mice was segmented using Amira (Thermo Fisher Scientific).

### Sholl Analysis and Measurement of Microglia Iba-1 Staining Intensity

Scanned images of Iba-1/TMEM119/DAPI-stained 10 μm thoracic spinal cord sections (*N* = 3–4/mouse, *N* = 5 mice/group) were opened in Zen (Zeiss). For each mouse, two frames having dimensions of 300 × 150 μm were captured in areas adjacent to sites of submeningeal inflammation in the anterior spinal cord white matter. Iba-1^+^/TMEM119^+^ microglia that had a DAPI^+^ nucleus were manually counted in each frame. The peak intensity of Iba-1 fluorescence for each microglia cell was measured in Zen by placing a circular region of interest (ROI) (5 μm^2^ in area) over each microglia cell and then subtracting the background fluorescence measured when the same ROI was placed adjacent to the cell. Sholl analysis of microglia was performed as follows. Images of individual Iba-1-stained microglia cells that were also verified to be TMEM119^+^ and have a DAPI^+^ nucleus were converted to 8-bit images, opened in Fiji, and then thresholded. Processes from surrounding microglia were deleted manually. The line segment tool was used to draw a line from the center of each soma to the longest process, which provided the maximum process length in μm and provided the region of interest for the Sholl analysis plugin. The Sholl analysis plugin in Fiji (36) was used to measure the intersections at each Sholl radius with the first shell set at 5 μm and subsequent shells set at 2 μm step sizes. This analysis provided the critical radius (radius value from the center of the cell with the highest intersections), the process maximum (the highest number of intersections, regardless of radius value), and the number of primary branches (estimated as the number of primary branches at the first shell). The soma size was measured after manual tracing using the polygon tool. Analyses were performed by an experimenter who was blinded to the identity of experimental groups.

### Flow Cytometric Analysis of CNS Mononuclear Cells

Mice were euthanized between 2 and 4 days after the onset of clinical symptoms and mice were matched for day of onset and clinical score. For BrdU incorporation assays, mice were also injected with 50 mg/kg BrdU i.p. for three consecutive days prior to cell isolation for flow cytometry. At endpoint, mice were perfused with 1 × PBS containing 5 U/ml heparin and mononuclear cells were isolated from the spinal cord and cerebellum as described above. For measurement of CD4^+^ T cell intracellular cytokine production, CNS mononuclear cells were incubated for 3.5 h at 37°C in complete RPMI that also contained 0.66 μl/ml GolgiStop™ (BD Bioscience) in the presence or absence of 10 ng/mL PMA (Sigma) and 535 ng/mL ionomycin (Cayman Chemical). To measure cytokine production by myeloid cells, cells were incubated for 5 h at 37°C in complete RPMI that contained GolgiStop™ (for IL-10 or IL-12p40) or GolgiPlug™ (BD Bioscience) (for IL-6) in the presence or absence of LPS (100 ng/mL, from *E. coli*, 055:B5, Sigma). Stimulated cells were centrifuged and washed twice in 1 × PBS prior to flow cytometry staining.

Flow cytometry staining for cell surface markers was done as described above using antibodies specific for the following markers (from ThermoFisher unless otherwise specified): B220 (RA3-6B2), CD4 (GK1.5), CD11b (M1/70), CD11c (N418), CD45 (30-F11), Gr-1 (RB6-8C5), Ly6G (1A8, BD Biosciences), I-A/I-E (M5/114.15.2, Biolegend). To assess intracellular cytokine production and levels of CD206 and iNOS, cells were fixed for 15 min with 4% PFA in 1 × PBS after staining for cell surface markers, washed, and then permeabilized in 1 × Perm/Wash Buffer (BD Bioscience) for 30 min prior to staining with relevant intracellular antibodies (from ThermoFisher unless otherwise specified): CD206 (C068C2, Biolegend), GM-CSF (MP1-22E9), IFN-γ (XMG1.2), IL-6 (MP5-20F3), IL-10 (JES5-16E3), IL-12p40 (C17.8), IL-17A (eBio17B7), iNOS (CXNFT), diluted in 1 × Perm/Wash buffer, washed twice in Perm/Wash, and resuspended in FACS buffer prior to flow cytometry acquisition. BrdU staining was conducted using the BrdU APC staining kit (BD Pharmingen). Data were analyzed using Flowjo (Flowjo LLC).

## Results

### Cultured Microglia That Are Deficient in PPAR-δ Exhibit Proliferation Defects and Are More Pro-inflammatory Upon Activation *in vitro* With LPS

Activation of microglia and the release of pro-inflammatory mediators by these cells is important in mediating neuroinflammation and tissue damage in EAE and MS (37). Previously, it was reported that PPAR-δ has a role promoting an M2-activated phenotype and in limiting a pro-inflammatory phenotype in macrophages in other tissues including the liver, adipose tissue, and spleen (16, 38, 39). To explore whether PPAR-δ also regulates microglia phenotype, we isolated primary microglia cells from mice that exhibit whole body deficiency in PPAR-δ (*Ppard*^−/−^) and *Ppard*^+/+^ controls and expanded these cells *in vitro* in the presence of M-CSF. The number and purity of these cells was assessed by counting and CD45 and CD11b staining (see representative flow cytometry plots in Supplementary Figure 1A). We first observed that the recovery of microglia was consistently lower in *Ppard*^−/−^ relative to *Ppard*^+/+^ microglia cultures (Supplementary Figure 1B). To gain insights into these growth deficits, after 2 passages, microglia were re-plated at a specific density and the proliferation of these cells to M-CSF was assessed by BrdU incorporation and CFSE-dilution assays (see representative staining in Supplementary Figure 1A). We observed that *Ppard*^−/−^ microglia exhibited reduced BrdU incorporation at 24 h, but not 48 h as compared to *Ppard*^+/+^ microglia (Supplementary Figure 1C). Compared to *Ppard*^+/+^ microglia, a lower frequency of *Ppard*^−/−^ microglia had divided by 48 h (Supplementary Figure 1D). In all cases, the viability and purity of microglia did not differ between *Ppard*^+/+^ and *Ppard*^−/−^ groups (Supplementary Figures 1E,F). Together, these data suggest that PPAR-δ-deficiency results in the compromised growth of microglia *in vitro* in response to M-CSF.

To evaluate whether PPAR-δ-deficiency also altered the pro-inflammatory potential of microglia, in separate studies, microglia were evaluated for the production of soluble mediators in response to stimulation with the Th1 cytokine IFN-γ alone or IFN-γ plus LPS (Supplementary Figure 1). Compared with *Ppard*^+/+^ microglia, *Ppard*^−/−^ microglia treated with IFN-γ secreted higher levels the pro-inflammatory lipid mediator prostaglandin E2 (Supplementary Figure 1H). When LPS was also added to the cultures, *Ppard*^−/−^ microglia also exhibited higher production of a number of other pro-inflammatory mediators than *Ppard*^+/+^ microglia including CXCL10, IL-6, TNF, RANTES, and nitric oxide as detected by Griess assay (Supplementary Figures 1G–L). There was also a tendency for IL-12p40 to be higher in the *Ppard*^−/−^ cultures (Supplementary Figure 1M). Therefore, consistent with the phenotype previously described for macrophages isolated from other tissues (16, 40), PPAR-δ-deficiency resulted in the enhanced production of certain pro-inflammatory mediators by microglia under M1-polarizing conditions.

### Microglia Deficiency of PPAR-δ Worsens the Severity of EAE

In light of these findings supporting a role for endogenous PPAR-δ activity in microglia, we next explored how PPAR-δ-deficiency in microglia impacts the development of EAE. For these studies, we acquired mice that express tamoxifen (TAM)-inducible Cre^ERT2^ fusion protein and eYFP under the endogenous *Cx3cr1* promoter (*Cx3cr1*^CreERT2^) (22). Upon administration of TAM, the *Cx3cr1*^CreERT2^ transgene is expressed in a number of myeloid cell types including microglia; however by 30 days post-TAM administration, expression of the transgene became largely restricted to microglia due to the more rapid turnover of the peripheral myeloid cell pool (Supplementary Figures 2A,B). To verify that expression of the *Cx3cr1*^CreERT2^ transgene induces efficient knockdown of *Ppard* in microglia, we crossed *Cx3cr1*^CreERT2^ mice with *Ppard* floxed mice to generate mice that were haplosufficient for *Cx3cr1*^CreERT2^ and homozoygous for the floxed allele (*Cx3cr1*^CreERT2^:*Ppard*^fl/fl^). We then evaluated *Ppard* expression in microglia in these mice and in *Ppard*^fl/fl^ controls at 60 days post-TAM treatment by real-time PCR. This experiment verified efficient knockdown of *Ppard* expression in *Cx3cr1*^CreERT2^:*Ppard*^fl/fl^ microglia compared to *Ppard*^fl/fl^ controls (Supplementary Figure 2C).

**Figure 1:**
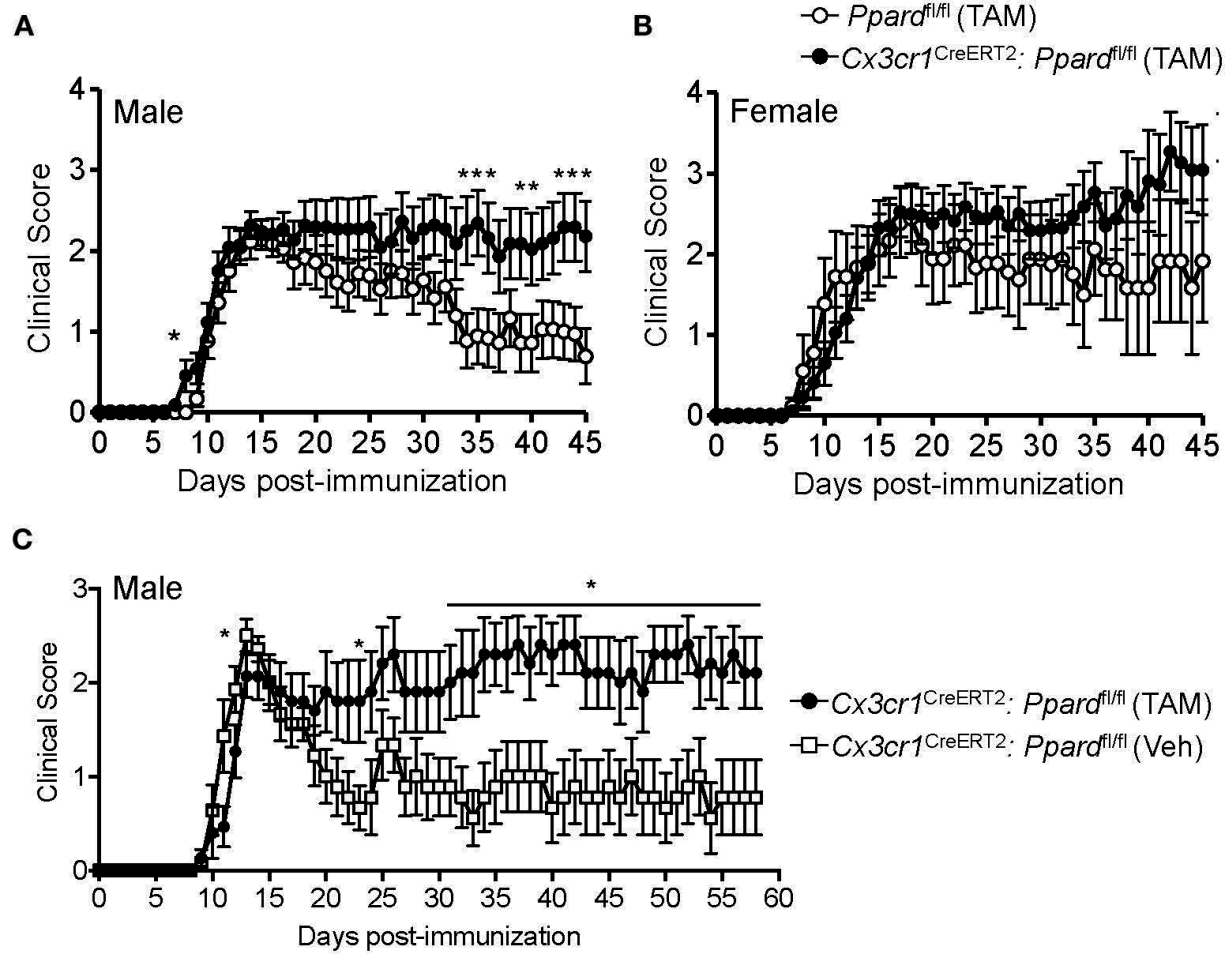
*Cx3cr1*^CreERT2^*:Ppard*^fl/fl^ male mice fail to recover in the post-acute phase of EAE. **(A,B)** EAE was induced in **(A)** male and **(B)** female *Ppard*^fl/fl^ or *Cx3cr1*^CreERT2^*:Ppard*^fl/fl^ mice (*n* = 9–14/group) at 30 days post-TAM treatment; mice were immunized with MOG p35-55 in CFA and given i.p. injections of pertussis toxin on days 0 and 2 post-immunization. Shown are the mean +/− SEM clinical scores of mice over 45 days. Results are from one experiment, but are representative of two that were performed per sex with similar sample sizes. **(C)** EAE was induced in male *Cx3cr1*^CreERT2^*:Ppard*^fl/fl^ mice that had been treated 30 days previous with TAM (*n* = 10) or with corn oil as a vehicle control (Veh) (*n* = 9). Mice were monitored for clinical score daily for 58 days. *Indicates a significant difference from *Ppard*^fl/fl^ control (*p* < 0.05) by Mann-Whitney two-tailed test. Results are from one experiment that was performed.

Next to explore whether PPAR-δ deficiency in microglia impacted the development or severity of EAE, we treated 6–8-week-old male and female *Cx3cr1*^CreERT2^:*Ppard*^fl/fl^ and littermate *Ppard*^fl/fl^ mice with TAM and 30 days later induced EAE by immunization with MOG p35-55/CFA and PTX. EAE initially presented with a similar onset and severity between *Cx3cr1*^CreERT2^:*Ppard*^fl/fl^ and *Ppard*^fl/fl^ mice (Figures 1A,B; Table 1); however, in the post-acute phase of EAE, male *Ppard*^fl/fl^ mice regained hindlimb function whereas clinical scores remained chronically elevated in the male *Cx3cr1*^CreERT2^:*Ppard*^fl/fl^ group (Figure 1A). The more severe EAE in male *Cx3cr1*^CreERT2^:*Ppard*^fl/fl^ mice was a result of higher peak scores, reduced hindlimb recovery, and higher deaths in this group (Table 1). A similar trend for higher clinical scores was apparent in female *Cx3cr1*^CreERT2^:*Ppard*^fl/fl^ mice, but this did not reach statistical significance (Figure 1B, Table 1).

**Table 1:** Clinical features of EAE in *Ppard*^fl/fl^ and *Cx3cr1*^CreERT2^*:Ppard*^fl/fl^ mice.

**Clinical feature**	***Ppard*^fl/fl^ male (TAM)**	***Cx3cr1*^CreERT^^2^*:Ppard*^fl/fl^ male (TAM)**	***Ppard*^fl/fl^ female (TAM)**	***Cx3cr1*^CreERT^^2^*:Ppard*^fl/fl^ female (TAM)**
Incidence	18/18	22/22	9/9	17/17
Day of onset	11.2 (0.4)	10.3 (0.5)	11.3 (1.2)	11.9 (0.8)
Peak score	2.8 (0.2)	3.4 (0.2)^a^	3.1 (0.4)	3.6 (0.3)
Cumulative score	53.0 (8.1)	78.7 (10.5)	62.8 (18.3)	83.7 (10.5)
# Mice remitting to score 0	16/18	8/22^b^	4/9	8/17
Death from disease	1/18	6/22	2/9	4/17

*These are data collected are pooled from two consecutive EAE experiments that were performed with similar results. Mice of both genotypes had been injected with tamoxifen (TAM) at 30 days prior to EAE induction. For day of onset, peak score, and cumulative score, values are mean + SEM*.

a *Significant from sex-matched Ppard^fl/fl^ control by two-tailed Mann-Whitney U test (P < 0.05)*.

b *Significant by Fisher exact test (P < 0.05)*.

In *Cx3cr1*^CreERT2^:*Ppard*^fl/fl^ mice, the *Cx3cr1*^CreERT2^ transgene replaces the wildtype *Cx3cr1* allele. Although *Cx3cr1* heterozygosity is reported not to influence EAE severity (41), given the importance of Cx3cr1 in microglia/neuronal interactions (42), we also compared EAE development between male *Cx3cr1*^CreERT2^:*Ppard*^fl/fl^ mice that had been treated 30 days previous with TAM or vehicle. In this system where mouse genotype was controlled, we observed similar results in that male *Cx3cr1*^CreERT2^*:Ppard*^fl/fl^ treated with TAM exhibited higher mean clinical scores in the post-acute phase of EAE compared to control *Cx3cr1*^CreERT2^*:Ppard*^fl/fl^ male mice treated with corn oil (Figure 1C and Table 2). The only difference in this experimental system was that TAM-treated mice exhibited a slight delay in EAE and a trend for reduced clinical scores compared to vehicle-treated mice in the acute phase (Figure 1C), which we found to associate with an unanticipated effect of the TAM in inhibiting myelin-specific Th17 responses and immune cell accumulation in the CNS (Supplementary Figure 3). Due to the anti-inflammatory effect of TAM, comparisons were made between TAM-treated *Cx3cr1*^CreERT2^:*Ppard*^fl/fl^ and *Ppard*^fl/fl^ mice in subsequent experiments.

**Table 2:** Clinical features of EAE in male *Cx3cr1*^CreERT2^*:Ppard*^fl/fl^ mice pre-treated with TAM or vehicle.

**Clinical feature**	***Cx3cr1*^CreERT^^2^*:Ppard*^fl/fl^ vehicle**	***Cx3cr1*^CreERT^^2^*:Ppard*^fl/fl^ TAM**
Incidence	9/9	10/10
Day of onset	11.0 (0.3)	11.7 (0.4)
Peak Score	2.8 (0.2)	2.9 (0.3)
Cumulative score	50.9 (14.8)	98.9 (14.9)^a^
# Mice remitting to score 0	7/9	3/9^b^
Death from disease	0/9	1/10

*Values are mean + SEM for day of onset, peak score, and cumulative score. Mice had been injected with tamoxifen (TAM) or vehicle (corn oil) as a control at 30 days prior to EAE induction*.

a *Significant from corn-oil treated control by two-tailed Mann-Whitney U test (P < 0.05)*.

b *Significant by Fisher exact test (P < 0.05)*.

### Th Cytokine Responses and CNS Immune Cell Infiltration Did Not Differ Between TAM-Treated *Cx3cr1*^CreERT2^:*Ppard*^fl/fl^ Mice in the Early Stages in EAE

**Figure 2:**
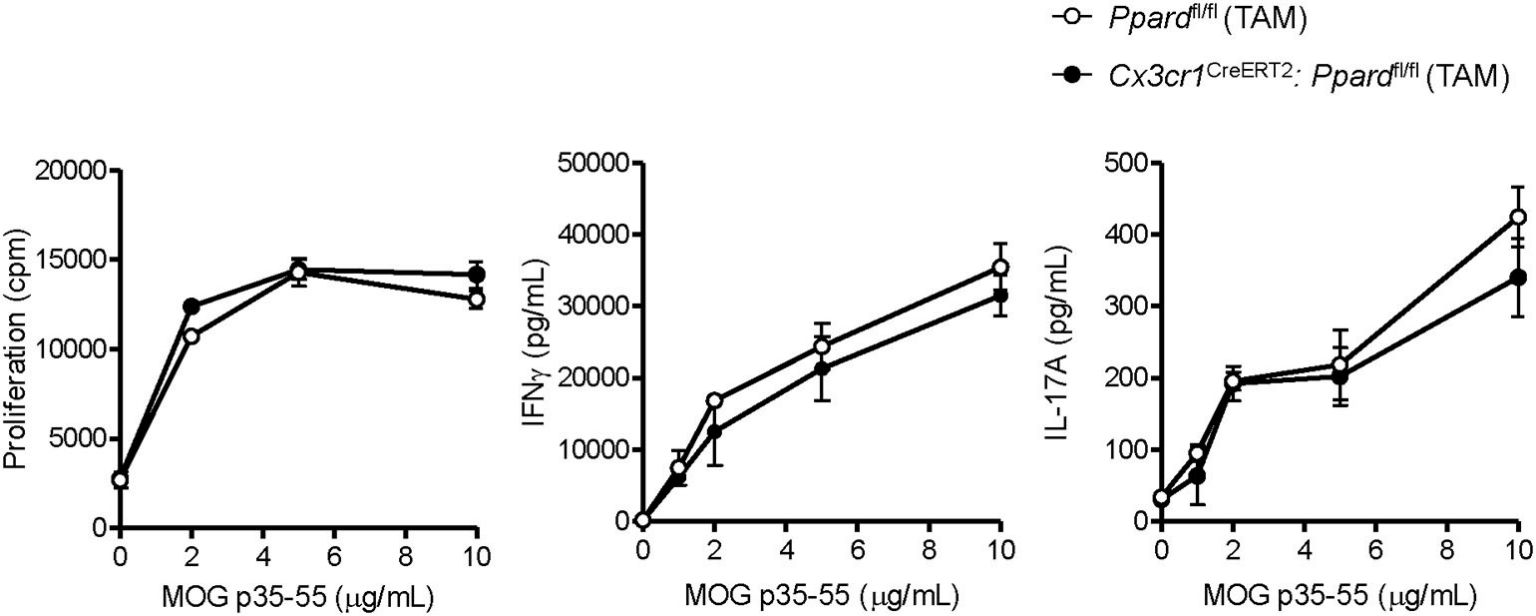
MOG p35-55-reactive T cell responses were not perturbed in the spleens of *Cx3cr1*^CreERT2^*: Ppard*^fl/fl^ mice. Spleens were isolated from MOG p35-55/CFA immunized male and female *Ppard*^fl/fl^ or *Cx3cr1*^CreERT2^*: Ppard*^fl/fl^ mice at day 9 post-immunization, were dissociated into a single cell suspension, and were stimulated *in vitro* with increasing concentrations of MOG p35-55. Proliferation of splenocytes (*left panel*) was measured using a [^3^H]-thymidine incorporation assay. Cells were pulsed with [^3^H]-thymidine at 48 h after stimulation with MOG p35-55 and cells were harvested 18 h later. Cpm, counts per minute. IFN-γ *(middle panel)* and IL-17A levels (*right panel*) were measured in culture supernatants by ELISA assay at 72 h of culture. Values are means ± SEM of levels seen in triplicate cultures of one experiment, but are representative of two independent experiments that were performed with similar results.

We previously reported that PPAR-**δ**-deficiency in CD11b^+^ dendritic cells (DC) enhances T helper cell priming during EAE (19). Since we observed that a low frequency of splenic DC expressed the *Cx3cr1*^CreERT2^ transgene (Supplementary Figures 2A,B), it remained possible that the severe EAE that developed in male *Cx3cr1*^CreERT2^:*Ppard*^fl/fl^ mice related to PPAR-**δ**–deficiency in DC. To rule this out, we examined MOG p35-55-specific T cell responses in *Cx3cr1*^CreERT2^:*Ppard*^fl/fl^ mice and *Ppard*^fl/fl^ mice at 9 days post-immunization with MOG p35-55/CFA. This experiment revealed no differences in the proliferation or Th cytokine production by spleen cells upon recall-stimulation with MOG p35-55 *ex vivo* (Figure 2). We also evaluated the number of immune cells in the CNS at 2-3 days post-onset of clinical signs (see Supplementary Figure 4 for representative gating strategy) and observed no significant differences in the numbers of total CD45^+^ leukocytes, monocytes/macrophages, neutrophils, DC, B cells, or total CD4^+^ T cells, nor in the frequencies of IFN-γ^+^, IL-17^+^, or GM-CSF^+^ CD4^+^ T cells between *Cx3cr1*^CreERT2^*:Ppard*^fl/fl^ and *Ppard*^fl/fl^ groups (Table 3). These findings reinforce the idea that the more severe EAE that developed in TAM-*Cx3cr1*^CreERT2^*:Ppard*^fl/fl^ mice was not driven by immune defects in the periphery and occurred downstream of immune cell infiltration in the CNS.

**Table 3:** Composition of immune cell infiltrate in the CNS early in EAE.

**Cell number (10^5^)**	***Ppard*^fl/fl^**	***Cx3cr1*^CreERT^^2^: *Ppard*^fl/fl^**	***P*-value**
Total leukocytes (CD45^+^ cells)	21.5 (3.0)	27.4 (4.0)	0.24
B cells (CD45^hi^B220^+^)	1.5 (0.2)	2.2 (0.4)	0.21
CD4^+^ T cells (CD45^+^CD4^+^)	1.8 (0.4)	2.9 (0.8)	0.27
Frequency of CD4^+^ T cells			
IFN-γ ^+^	16.2 (2.1)	17.0 (2.6)	0.83
IL-17^+^	2.4 (0.8)	2.0 (0.3)	0.63
GM-CSF^+^	2.4 (0.5)	2.8 (0.7)	0.64
Neutrophils (CD45^hi^CD11b^+^Ly6G^+^)	8.0 (2.6)	8.9 (1.4)	0.78
Monocytes/Macrophages (CD45^hi^Ly6G^−^CD11b^+^CD11c^−^)	4.1 (0.9)	5.2 (0.5)	0.31
Dendritic cells (CD45^hi^Ly6G^−^CD11b^+^CD11c^+^)	3.7 (0.8)	5.0 (2.2)	0.62
Microglia (CD45^lo^CD11b^+^)	0.4 (0.1)	0.4 (0.1)	0.87
Other CD45^hi^	2.0 (0.3)	2.8 (0.3)	0.11

*Values are mean (SEM) of N = 4–6 mice/group. Data are from one experiment performed in male mice and are representative of two that were performed. T cells were harvested from the spinal cord and cerebellums of mice at 3 days post onset of first clinical symptoms of EAE. Mice were matched for clinical score and disease duration*.

### *Cx3cr1*^CreERT2^: *Ppard*^fl/fl^ Mice Exhibited More Severe Inflammation and Tissue Loss Later in EAE

**Figure 3:**
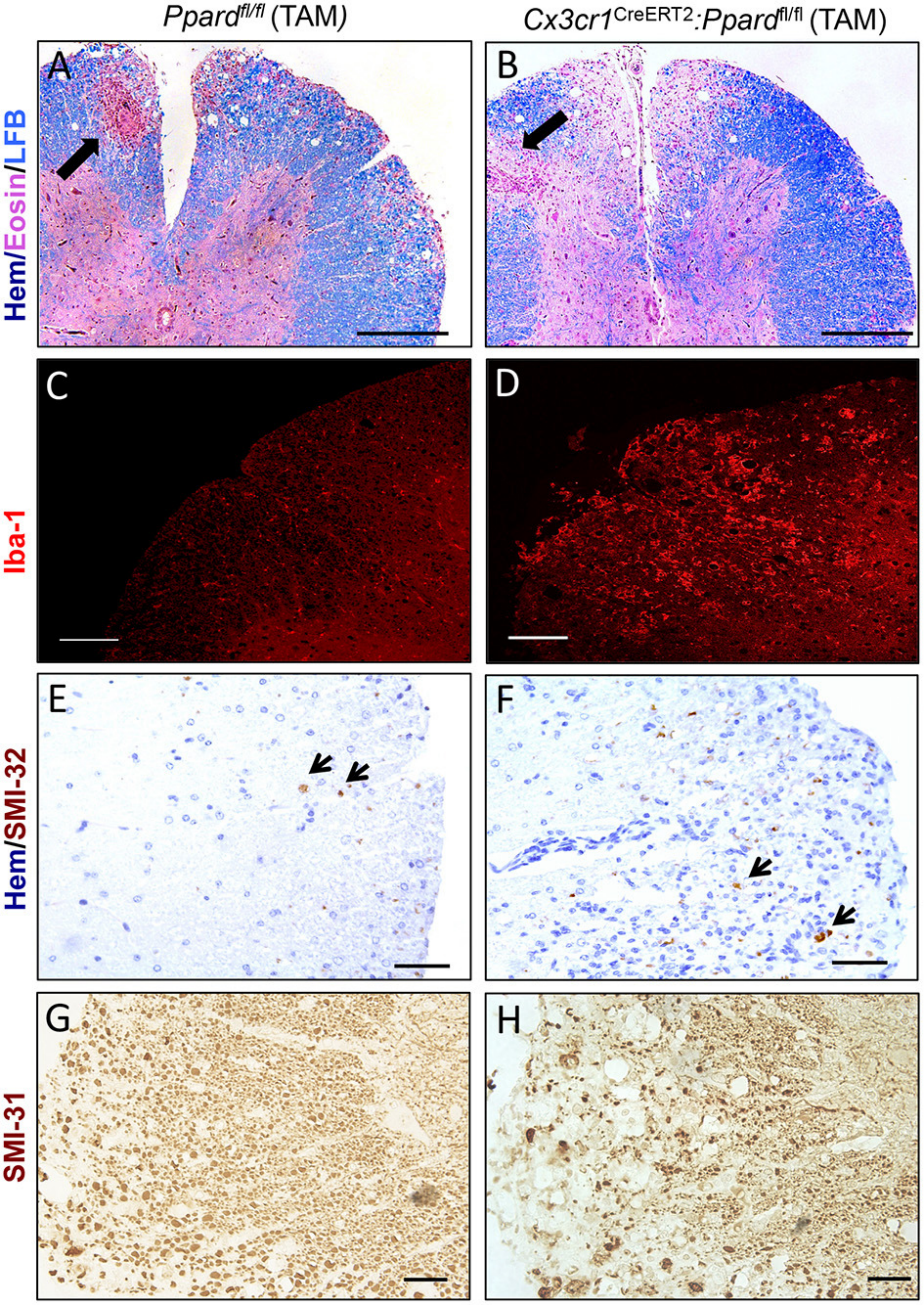
Representative staining of inflammation, myelin, and non-phosphorylated and phosphorylated neurofilament heavy in axons in the spinal cords of *Cx3cr1*^CreERT2^*:Ppard*^fl/fl^ mice and *Ppard*^fl/fl^ mice at 45 days of EAE. Spinal cords were dissected from male *Cx3cr1*^CreERT2^*:Ppard*^fl/fl^ and *Ppard*^fl/fl^ mice with representative clinical scores of the group were preserved in formalin, embedded in paraffin, and cut in cross-section (5 μm). Images show representative staining for Hematoxylin (Hem), Eosin, and Luxol fast blue (LFB) triple stain (Scale bar = 100 μm, thick arrows point to perivascular cuff) **(A,B)**, Iba-1 immunofluorescence (Scale bar = 80 μm) **(C,D)**, SMI-32 staining for non-phosphorylated neurofilament heavy (small arrows point to SMI-32^+^ axons, Scale bar = 20 μm) **(E,F)**, and SMI-31 staining for phosphorylated neurofilament heavy (Scale bar = 50 μm) **(G,H)**.

**Figure 4:**
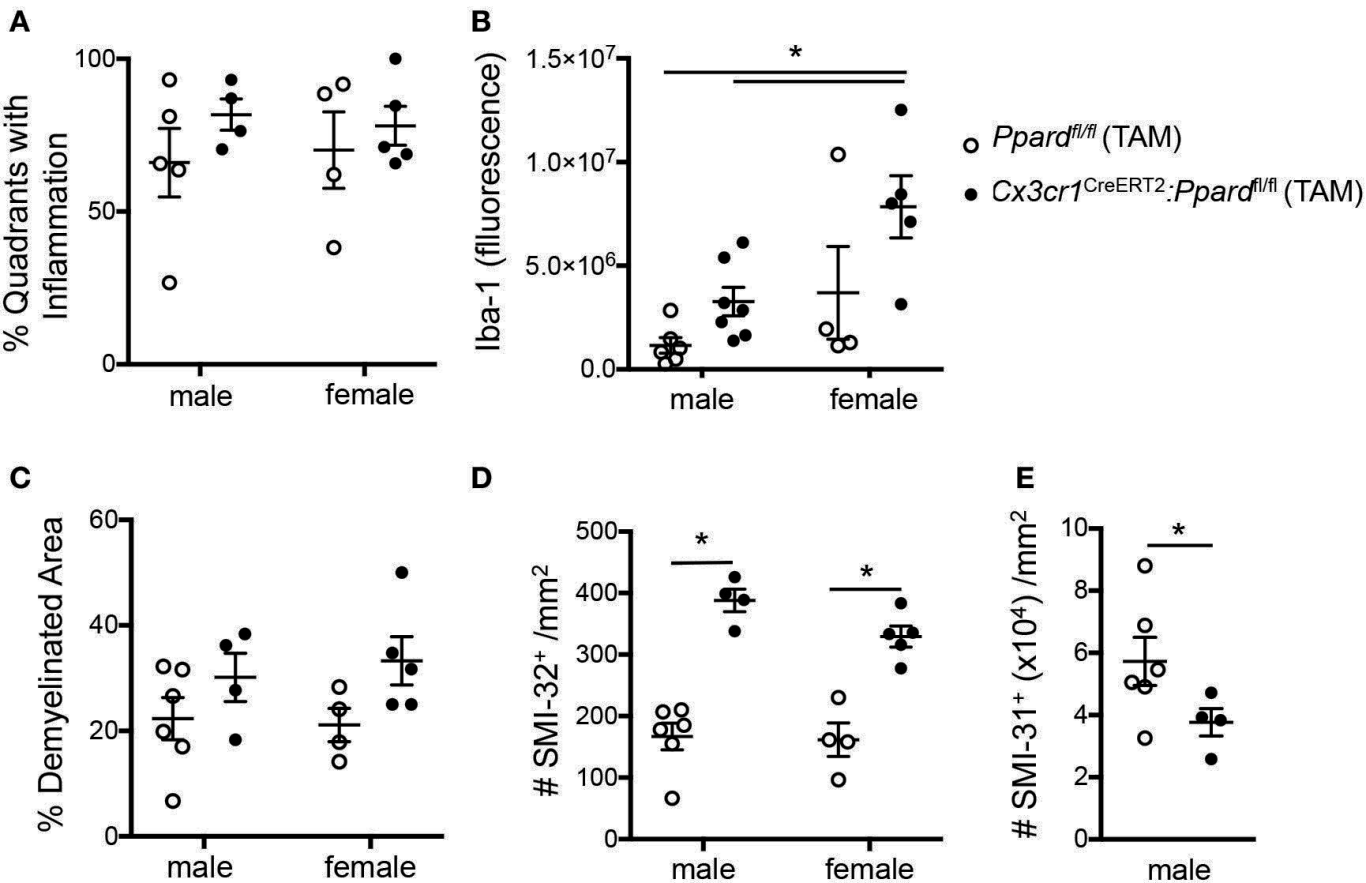
*Cx3cr1*^CreERT2^*:Ppard*^fl/fl^ mice exhibit increased Iba-1 staining intensity and axon injury and loss at 45 days of EAE compared to *Ppard*^fl/fl^ mice. Spinal cords were dissected from male and female *Cx3cr1*^CreERT2^*:Ppard*^fl/fl^ and *Ppard*^fl/fl^ mice with representative clinical scores of the group after 45 days of EAE and were preserved in formalin for histopathological analyses. Spinal cord sections were cut in cross-section (10–12 per mouse, sampled throughout the cord), were stained with Hematoxylin &Eosin/Luxol fast blue, Iba-1 immunofluorescence, SMI-32 to label injured axons (non-phosphorylated neurofilament heavy), or SMI-31 to label phosphorylated neurofilament heavy (all axons). **(A)** Percent of spinal cord quadrants that contained inflammatory/demyelinating lesions (10–12 sections sampled per mouse, *N* = 4–7/group). **(B)** Iba-1 section immunofluorescence detected at the level of the thoracic cord, corrected for background fluorescence (*N* = 2–3 sections/mouse, *N* = 4–7 mice/group). **(C)** Percent demyelination in white matter, which was determined by measuring white matter area with myelin pallor in 10–12 sections of the cord/mouse (*N* = 4–7 mice/group) and dividing this by total white matter area sampled. **(D)** Number of SMI-32^+^ axons/mm^2^ in these same sections. **(E)** Number of SMI-31^+^ axons/mm^2^ at the level of the thoracic cord in male mice (*N* = 4 areas/section, 2–3 sections/mouse, N-4-6 mice/group). Values in graphs are means ± SEM of values of mean values obtained in individual mice. In A-D, *denotes a significant difference at *p* < 0.05, as determined by Tukey *post-hoc* test. In E, *denotes a significant difference at *p* < 0.05 as determined by Mann-Whitney *U* test.

To gain insights into the cause of the more severe EAE phenotype of *Cx3cr1*^CreERT2^:*Ppard*^fl/fl^ mice, we conducted a histological analysis of inflammation, demyelination, and neuronal injury in the spinal cords of *Cx3cr1*^CreERT2^:*Ppard*^fl/fl^ and *Ppard*^fl/fl^ mice at the endpoint of EAE. For this analysis, spinal cord sections were stained with H&E and LFB to reveal inflammatory demyelinating lesions (Figures 3A,B), Iba-1 to stain infiltrating monocytes and microglia (Figures 3C,D), SMI-32 to stain injured axons (Figures 3E,F), and SMI-31 to label all axons (Figures 3G,H). We observed no significant difference between groups in the percent of spinal cord quadrants that had inflammatory lesions present (Figure 4A), however the intensity of total Iba-1 staining as measured by total section fluorescence was higher in *Cx3cr1*^CreERT2^*:Ppard*^fl/fl^ compared to *Ppard*^fl/fl^ mice [2-way ANOVA genotype effect, *F*_(1,18)_ = 7.217] (Figure 3D vs. Figure 3C, Figure 4B). Iba-1 staining intensity was also higher overall in the female groups (2-way ANOVA sex effect, *F*_(1,18)_ = 9.289 *P* = 00069), potentially explaining why female *Ppard*^fl/fl^ mice, unlike male counterparts, failed to remit in the post-acute phase of EAE.

To investigate tissue damage in EAE, we calculated the percent demyelinated area on LFB-stained sections and the number of injured SMI-32^+^ axons in the spinal cord white matter. This analysis revealed that *Cx3cr1*^CreERT2^:*Ppard*^fl/fl^ mice, regardless of sex, exhibited a higher percentage of white matter that was demyelinated [2-way ANOVA, genotype effect, *F*_(1, 15)_ = 5.44, *P* = 0.03] (Figure 4C) as well as a strikingly higher number of injured axons [2-way ANOVA, genotype effect *F*_(1, 15)_ = 79.41, *P* < 0.0001] (Figure 4D) relative to *Ppard*^fl/fl^ groups. To evaluate whether axon numbers were also decreased in *Cx3cr1*^CreERT2^:*Ppard*^fl/fl^ mice vs. *Ppard*^fl/fl^ mice, we evaluated the density of SMI-31^+^ axons in the male *Cx3cr1*^CreERT2^:*Ppard*^fl/fl^ and *Ppard*^fl/fl^ mice at the level of the thoracic cord. This analysis revealed lowered axon density in the *Cx3cr1*^CreERT2^:*Ppard*^fl/fl^ group (Figure 4E). Therefore, microglial deficiency in PPAR-δ resulted in increased inflammation and axon injury during EAE. The finding that the this phenotype was apparent in both male and female *Cx3cr1*^CreERT2^:*Ppard*^fl/fl^ mice suggested that it was not sex-dependent. Accordingly, subsequent analyses focused on the male sex.

**Figure 5:**
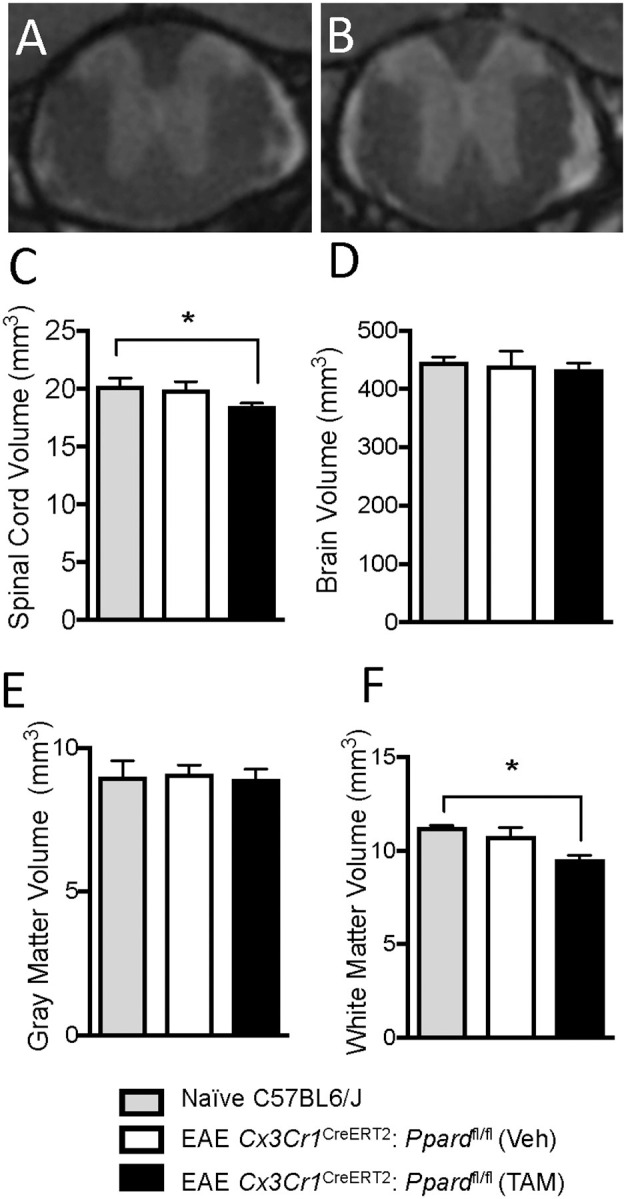
*Cx3cr1*^CreERT2^*:Ppard*^fl/fl^ mice exhibit spinal cord atrophy during EAE. EAE was induced in male *Cx3cr1*^CreERT2^*:Ppard*^fl/fl^ mice at 30 days post-injection with TAM or corn oil vehicle (Veh). Mice were monitored for clinical symptoms for 58 days after which mice were perfused with fixative containing Gadolinium contrast agent. Then brain and spinal cords of representative *Cx3cr1*^CreERT2^*:Ppard*^fl/fl^ mice treated with TAM (*N* = 8/group) or Veh (*N* = 6/group) along with age-and sex-matched control mice without EAE (*N* = 3/group) were preserved for volumetric MR analysis of the thoracic (T1-T7) spinal cord and brain. **(A,B)** Shows representative MR images of the thoracic spinal cord in Veh **(A)** or TAM-treated **(B)** *Cx3cr1*^CreERT2^*:Ppard*^fl/fl^ mice. **(C)** Total spinal cord volume, **(D)** total brain volume, **(E)** spinal cord gray matter volume, and **(F)** spinal cord white matter volume in control mice and in the EAE groups. Values are means + SEM of individual mice. *denotes a significant different from the non-EAE control (*p* < 0.05) as determined by Kruskal-Wallis test followed by a Dunn's multiple comparison test.

To evaluate whether the demyelination and neuronal injury resulted in tissue loss, we also measured the volume of the thoracic spinal cord in mice with representative clinical scores at 59 days of EAE (experiment in Figure 1C) and of age- and sex-matched controls that did not have EAE. For this experiment, mice were perfused with fixative containing gadolinium contrast at endpoint and high-resolution T2-weighted MRI scans were obtained from fixed whole brain and the thoracic spinal cord T1-T7 segment (see representative images in Figures 5A,B). We observed a decrease in total spinal cord volume as well as white matter, but not gray matter volume in *Cx3cr1*^CreERT2^*:Ppard*^fl/fl^ mice treated with TAM compared with naïve control mice that did not have EAE (Figures 5C,E,F). This was accompanied by the appearance of a hyper-intense rim of contrast around the spinal cord (Figure 5B), which we anticipate is due to the accumulation of contrast agent in the increased space around the spinal cord post-fixation. These findings of more extensive inflammation, axon injury, and tissue loss in the spinal cord further explain why TAM-treated male *Cx3cr1*^CreERT2^:*Ppard*^fl/fl^ mice failed to regain hindlimb function in the post-acute phase of EAE.

Though inflammation predominates in the spinal cord in EAE, lesions can be detected in certain white matter tracts and under the meninges and in the ventricles of the brain. We therefore assessed total brain volume and conducted a volumetric analysis of 62 individual brain regions in TAM- or vehicle-treated *Cx3cr1*^CreERT2^*:Ppard*^fl/fl^ mice. Though total brain volume was not significantly lowered at 60 days of EAE in either group relative to age-matched mice without EAE (Figure 5D), 16 brain regions were significantly atrophied selectively in the TAM-treated *Cx3cr1*^CreERT2^*:Ppard*^fl/fl^ group. These regions included structures in the hippocampus, the medial and lateral septum, the striatum, the optic tract, and white matter tracts in the midbrain that run in close proximity to the hippocampus or third ventricle (Supplementary Table 1).

Inspection of the pathology of the brains of mice revealed that microglia in the *Cx3cr1*^CreERT2^*:Ppard*^fl/fl^ group had a more reactive appearance with increased Iba-1 staining in some of the regions found to be smaller on MRI, including those that were infiltrated by immune cells (cerebellum and brain stem) as well as structures that were in close proximity to sites of meningeal (olfactory tubercle) or intraventricular inflammation (hippocampus and septum) (Supplementary Figure 5).

### Microglia in *Cx3cr1*^CreERT2^:*Ppard*^fl/fl^ Mice Exhibit a More Re-active Phenotype Early in EAE

When microglia become active, they upregulate the expression of Iba-1, retract their processes, and adopt a more amoeboid morphology (43). Since inflammation had escalated in *Cx3cr1*^CreERT2^*:Ppard*^fl/fl^ mice in the chronic phase of EAE (Figures 4A,B), it was difficult to interpret whether PPAR-δ-deficiency was driving increased microglia reactivity or that PPAR-δ-deficient microglia appeared more reactive because of the enhanced CNS inflammation. Subsequent studies were therefore designed to evaluate microglia activation state at 2–3 days post-onset of EAE, a time when the spinal cord was equivalently infiltrated by leukocytes (Table 3) and when clinical scores were equivalent between *Cx3cr1*^CreERT2^*:Ppard*^fl/fl^ and *Ppard*^fl/fl^ groups (Figure 1, Table 4).

**Table 4:** Microglia Iba-1 staining intensity and morphology in the spinal cord of *Cx3cr1*^CreERT2^*:Ppard*^fl/fl^ and *Ppard*^fl/fl^ mice at 2-3 days post-onset of EAE.

**Measure**	***Ppard*^fl/fl^ (TAM)**	***Cx3cr1*^CreERT2^*:Ppard*^fl/fl^ (TAM)**	***P-*value**
Clinical score	2.5 (0.1)	2.6 (0.2)	0.45
Number of microglia	13.9 (0.4)	18.7 (2.1)	0.02*
Iba-1 intensity	13065 (768)	14408 (938)	0.30
Maximum branch length (μm)	14.9 (1.3)	11.6 (0.6)	0.049*
Soma area (μm^2^)	56.7 (2.5)	61.8 (5.0)	0.40
Process maximum (N_m_)	3.2 (0.2)	2.9 (0.2)	0.27
Critical radius (μm)	7.2 (0.9)	6.1 (0.2)	0.13
Number of primary branches (N_p_)	2.6 (0.4)	2.2 (0.1)	0.51

Mice in each group were treated to TAM at 35 days prior to EAE induction. Mice were scored for clinical symptoms and were sacrificed at 2-3 days post-onset of EAE. Thoracic spinal cord samples were fixed and cross-sections (10 μm) were cut (2-3 sections/mouse) and stained with Iba-1 and TMEM119 antibodies and DAPI. For each mouse, two fields were captured in lesioned areas of the anterior thoracic cord. Microglia (TMEM119^+^Iba-1^+^DAPI^+^) cells were counted in these fields and Iba-1 staining intensity was measured. Sholl analysis was performed on Iba-1^+^TMEM119^+^DAPI^+^ microglia (N = 15 cells/mouse) using the ImageJ plugin. Values are mean + SEM of the average values obtained from individual mice (N = 5 mice/group). The p values are marked with an asterisk (*) if the difference between Cx3cr1^CreERT2^:Ppard^fl/fl^ and Ppard^fl/fl^ were significantly different by two-tailed T-test, N = 5 mice per group. Data are from one independent experiment.

To evaluate microglia reactivity, we isolated spinal cords from *Ppard*^fl/fl^ mice and *Cx3cr1*^CreERT2^*:Ppard*^fl/fl^ mice and co-stained thoracic sections with microglia markers Iba-1 and TMEM119 and DAPI (representative staining in Supplementary Figure 6). We then captured images of microglia in inflamed regions of the anterior spinal cord and counted microglia, measured cellular Iba-1 fluorescence, and assessed the morphology of microglia (in the Iba-1 channel) in cells that were also TMEM119^+^ and had a DAPI^+^ nucleus. This analysis revealed that microglia from *Cx3cr1*^CreERT2^*:Ppard*^fl/fl^ mice were more densely represented in inflamed areas and had a significantly shorter maximum branch length compared to those observed in spinal cords of *Ppard*^fl/fl^ mice. Similarly, there was a trend for a smaller critical radius in microglia of *Cx3cr1*^CreERT2^*:Ppard*^fl/fl^ mice, which is the distance from the microglia center, where most Sholl intersections are observed. Iba-1 staining intensity and other measures of microglia morphology were not significantly different between groups. Together, these results suggest that microglia in *Cx3cr1*^CreERT2^*:Ppard*^fl/fl^ mice exhibited some features consistent with a more reactive phenotype in EAE.

### Microglia in *Cx3cr1*^CreERT2^*:Ppard*^fl/fl^ Mice Did Not Adopt a More “M1 Phenotype” Nor Exhibited Impaired Proliferation Early in EAE

**Figure 6:**
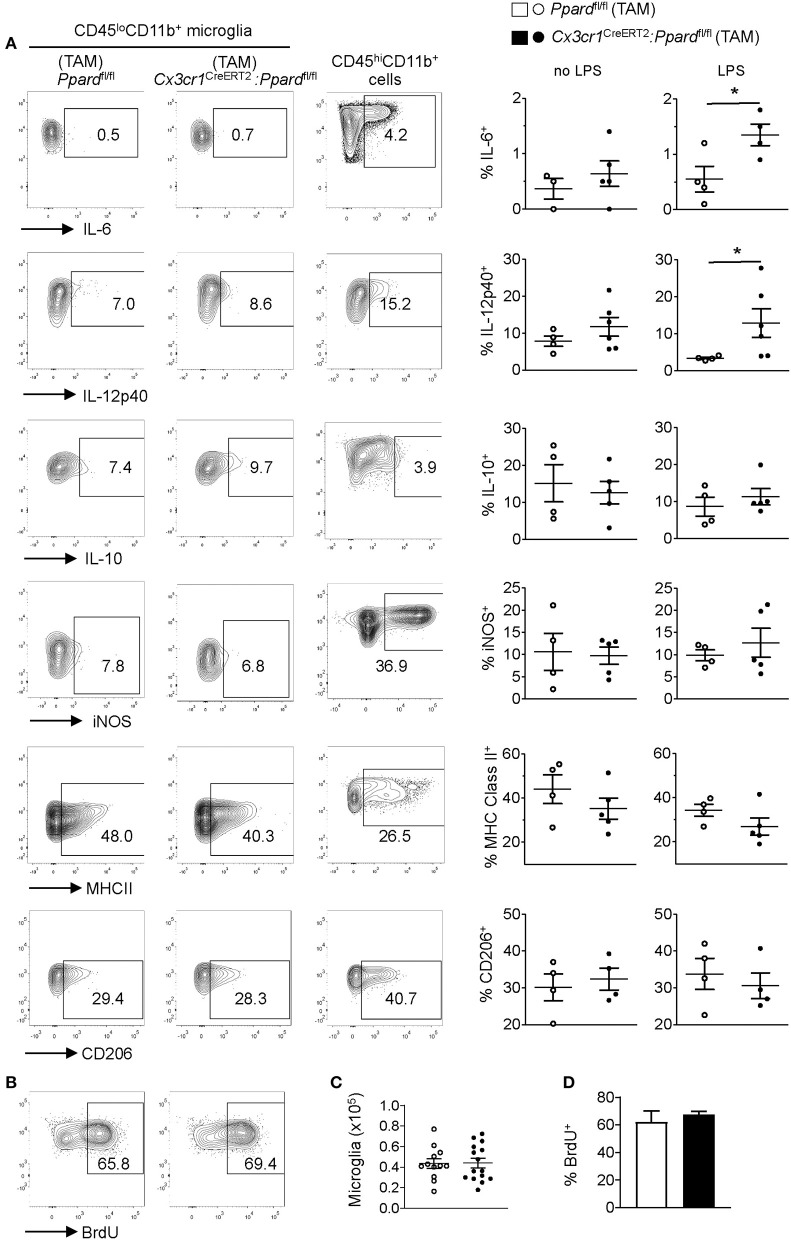
*Cx3cr1*^CreERT2^*:Ppard*^fl/fl^ mice microglia do not exhibit an M1 phenotype or defective proliferation *in vivo* during EAE. EAE was induced in mice and at several days after the onset of clinical signs. Mice (*N* = 4 *Ppard*^fl/fl^ and *N* = 5 *Cx3cr1*^CreERT2^*:Ppard*^fl/fl^) were killed and CNS mononuclear cells were isolated from the spinal cord and cerebellum (tissue was pooled for analysis). The expression of IL-6, IL-12p40, IL-10, iNOS, MHC Class II, and CD206 was examined in microglia (CD45^lo^CD11b^+^Ly6G^−^) that were gated based on fluorescence minus one controls. [**(A)**, left and middle panels]. Positive control staining consisted of staining in monocyte/macrophages (CD45^hi^CD11b^+^CD11c^−^Ly6G^−^) in the same sample [**(A)**, right panels]. Y-axis in all plots are either FSC-A or CD11b staining. Graphs on the right show the mean + SEM percent of cells staining positive for the indicated marker. Symbols represent individual mice. Values in A are from one representative experiment of three that were performed. **(B)** Representative BrdU staining in microglia (gated on live CD45^lo^CD11b^+^Ly6G^−^) cells in *Ppard*^fl/fl^ (*left panel*) and *Cx3cr1*^CreERT2^*:Ppard*^fl/fl^ (*right panel*) groups. **(C)** Mean number of microglia isolated from the CNS (total mononuclear cells x percent microglia gate). **(D)** Mean + SEM BrdU^+^ microglia. Values in **(C,D)** were pooled from two consecutive experiments with similar results, *n* = 12 *Ppard*^fl/fl^ and *n* = 15 *Cx3cr1*^CreERT2^*:Ppard*^fl/fl^ mice.

Influenced by our *in vitro* studies of microglia (Supplementary Figure 1), we next explored whether the more severe EAE observed in *Cx3cr1*^CreERT2^*:Ppard*^fl/fl^ mice related to shift toward higher microglia production of M1-associated or pro-inflammatory mediators. We therefore evaluated specific M1/pro-inflammatory (Nos2, MHC Class II, IL-12p40, IL-6) and M2/anti-inflammatory markers (CD206 and IL-10) on CD45^lo^CD11b^+^ microglia by flow cytometry in *Cx3cr1*^CreERT2^*:Ppard*^fl/fl^ and *Ppard*^fl/fl^ mice at 2–4 days post-onset of EAE. Cytokine expression was evaluated by intracellular staining after 5 h of culture with golgi inhibitors in the presence or absence of LPS. Assessment of M1 markers on CD45^hi^CD11b^+^ cells (monocytes/macrophages/neutrophils) provided a positive control for M1 marker staining (Figure 6A).

Consistent with our findings in cultured microglia, we observed that a higher frequency of *Cx3cr1*^CreERT2^*:Ppard*^fl/fl^ microglia produced IL-12p40 and IL-6 upon LPS stimulation compared to *Ppard*^fl/fl^ controls (Figure 6A, *right panels*). However, when LPS was not added to the cultures the cytokine profile and expressions of other M1 and M2 markers was indistinguishable between *Cx3cr1*^CreERT2^*:Ppard*^fl/fl^ and *Ppard*^fl/fl^ groups (Figure 6A). Therefore, while PPAR-δ does regulate the expression of IL-6 and IL-12p40 downstream of LPS stimulation, these cytokines were not differentially expressed by microglia during EAE.

In light of our observation of slowed microglia growth *in vitro* with PPAR-δ-deficiency (Supplementary Figure 1) and increased microglia numbers in inflamed regions (Table 4), we also evaluated the proliferation of microglia in the CNS during EAE by *in vivo* BrdU incorporation assay (44) (see representative staining in Figure 6B). This analysis revealed no difference between groups in either the total number of CD45^lo^CD11b^+^ microglia isolated from the spinal cord and cerebellum (Figure 6C) or the frequency of microglia that took up BrdU over the first 3 days of EAE (Figures 6B,D). Therefore, neither enhanced M1 programming nor defects in microglia proliferation were the cause of the more extensive inflammation or tissue damage in *Cx3cr1*^CreERT2^*:Ppard*^fl/fl^ mice during EAE.

### Gene Profiling of PPAR-δ-Deficient Microglia Revealed a Greater Shift Toward a Disease-Associated Microglia (DAM) Phenotype in EAE

To better understand the basis of the more reactive microglia phenotype we conducted an analysis of the transcriptome of CD45^lo^CD11b^+^ microglia sorted from TAM-treated *Cx3cr1*^CreERT2^*:Ppard*^fl/fl^ and *Ppard*^fl/fl^ mice at 2–3 days post-onset of EAE. For this analysis, spinal cords were isolated from mice (*N* = 9 mice/group) that were matched for both disease duration and clinical scores. Samples were pooled (*N* = 3 mice/sample, three samples per group) to increased microglia yields for RNA sequencing studies. Only samples that passed quality control (*N* = 2/genotype) were submitted for sequencing.

**Figure 7:**
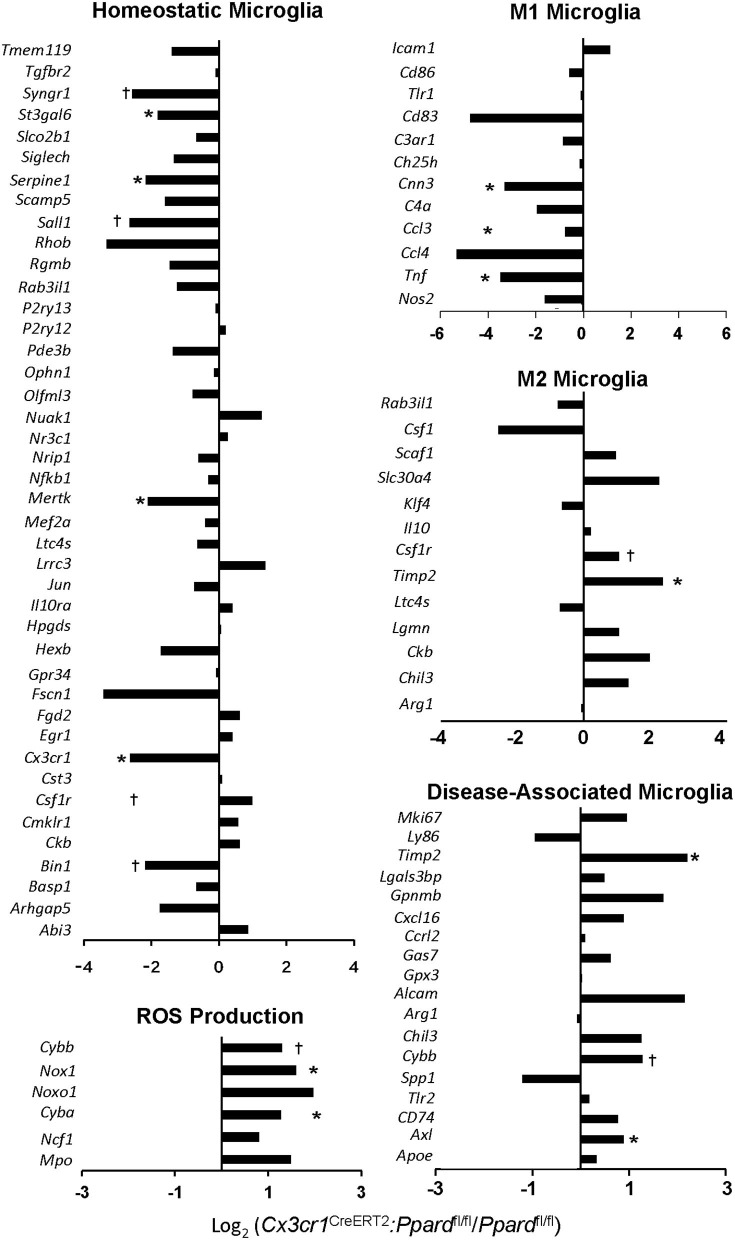
*Cx3cr1*^CreERT2^*:Ppard*^fl/fl^ mice show reduced expression of certain homeostatic microglia genes and a skew towards a M2/neurodegenerative microglia phenotype. *Ppard*^fl/fl^ and *Cx3cr1*^CreERT2^*:Ppard*^fl/fl^ were treated with TAM or vehicle and 60 days later, EAE was induced with MOG p35-55/CFA + PTX. Between 2 and 4 days post-onset, spinal cords were isolated from TAM-treated *Ppard*^fl/fl^ and *Cx3cr1*^CreERT2^*:Ppard*^fl/fl^ mice that were matched for disease duration and clinical scores. Spinal cords from individual mice (*N* = 9/group) were pooled (*N* = 3 mice/sample) for mononuclear cell isolation and FACS-sorting to generate *N* = 3 pooled samples per group. Two of the three samples that passed RNA quality control were submitted for RNA sequencing. Bar graphs show the log_2_ (fold change of FPKM values from *Cx3cr1*^CreERT2^*:Ppard*^fl/fl^ samples divided by FPKM values of the *Ppard*^fl/fl^ samples). Genes expressed at lower levels in *Cx3cr1*^CreERT2^*:Ppard*^fl/fl^ mice are negative and those expressed at higher levels are positive. *Indicates genes that showed a difference between *Ppard*^fl/fl^ and *Cx3cr1*^CreERT2^*:Ppard*^fl/fl^ by two-tailed Mann-Whitney test at the *P* = 0.05 level (*) or *P* = 0.1 level (†). Gene lists were compared against those described in (11, 45) and only genes with FPKM values in both of the *Ppard*^fl/fl^ samples are shown. For the case of genes with decreased expression in *Cx3cr1*^CreERT2^*:Ppard*^fl/fl^ some samples had 0 FPKM values. In this case, a value of 0.1 was assigned for the sample to permit the calculation of log_2_(fold-change).

First, we compared the expression of genes that describe a “homeostatic,” “M1,” “M2,” and DAM microglia phenotype between *Cx3cr1*^CreERT2^*:Ppard*^fl/fl^ and *Ppard*^fl/fl^ groups (11, 45). Consistent with our *in situ* findings of shorter branch length in microglia in *Cx3cr1*^CreERT2^*:Ppard*^fl/fl^ mice during EAE, we observed significantly lowered expression of several genes associated with a homeostatic microglia phenotype in the *Cx3cr1*^CreERT2^*:Ppard*^fl/fl^ group (Figure 7). Interestingly, several M1 signature genes exhibited lowered expression in *Cx3cr1*^CreERT2^*:Ppard*^fl/fl^ microglia. We also observed a tendency for higher expression of specific genes that describe an M2 or DAM signature in the *Cx3cr1*^CreERT2^*:Ppard*^fl/fl^ microglia. Specifically, we observed upregulation of *Axl*, that encodes a TAM receptor tyrosine kinase that is upregulated in EAE and mediates phagocytosis of neuronal debris (11, 46), *Timp2*, which is an inhibitor of matrix metalloproteinases (MMPs), as well as higher expression of components of the NADPH oxidase complex (*Nox1, Cyba*), which is a major producer of intracellular and extracellular ROS (11, 45). ROS are known contribute to neuronal injury in EAE (14).

To gain additional insights into PPAR-δ-dependent regulation of microglia gene expression, we examined the functions of individual differentially-expressed genes (DEGs) between *Cx3cr1*^CreERT2^*:Ppard*^fl/fl^ and *Ppard*^fl/fl^ microglia (Supplementary Tables 2, 3) and conducted DAVID functional annotation analysis of DEGs (Supplementary Tables 4, 5). We detected higher expression of genes in *Cx3cr1*^CreERT2^*:Ppard*^fl/fl^ microglia that sit in pathways downstream and upstream of NADPH activation. This included genes encoding High mobility box protein 1 (*Hbp1*) and complement receptor 3 (*C3*), which serve as components of a damage-sensing pathway that can activate NADPH oxidase (47, 48), and genes in the PI3K/Akt pathway (*Pik3cd, Akt2, Rs6k*) (49). Consistent with these findings, *Cx3cr1*^CreERT2^*:Ppard*^fl/fl^ microglia also exhibited a gene signature reflecting increased oxidative stress. Specifically, we detected higher expression of cellular and mitochondrial chaperonins (*Hspa14, Dnajc3, Grpel1, Pfdn2*) and genes that are known to be upregulated or involved in the response to oxidative stress or repair of oxidatively-damaged proteins (*Msra, Sirt1, Foxo3*) (50) (Supplementary Table 2). Two genes with anti-oxidant functions in mitochondria (*Gss, Trap1)* were expressed at decreased levels in *Cx3cr1*^CreERT2^*:Ppard*^fl/fl^ microglia (Supplementary Table 3).

Mitochondria, once damaged by oxidative stress initially compensate by upregulating the expression of mitochondrial enzymes (51). When mitochondria fail to meet energy demands, these organelles are disposed of via a specialized form of autophagy, called mitophagy (51). In this regard, we noted that PPAR-δ-deficient microglia exhibited higher expression of genes encoding enzymes in the citric acid cycle (*Dlst, Me2*) and oxidative phosphorylation (*Cox15, Ndufb10, Uqcrb, Atp5h*) (Supplementary Table 2). Furthermore, “Mitophagy in response to mitochondrial depolarization” (GO: 0098779) was the most significantly downregulated biological process in *Cx3cr1*^CreERT2^*:Ppard*^fl/fl^ microglia (Supplementary Table 5).

Relevant to the finding of a more amoeboid microglia morphology in spinal cords of *Cx3cr1*^CreERT2^*:Ppard*^fl/fl^ mice, we observed an upregulation of the biological process of microtubule polymerization (GO:031116) (Supplementary Table 4). Another process that was significantly higher in *Cx3cr1*^CreERT2^*:Ppard*^fl/fl^ mice was lipid transport (GO: 0006869) and this pathway included genes involved in the uptake, intracellular transport and oxidation of lipids in mitochondria (*Prelid1, Apobr, Lbp, Mttp, Scp2, Tspo*) (Supplementary Table 4). This together with the increased *Axl* expression suggested that *Cx3cr1*^CreERT2^*:Ppard*^fl/fl^ microglia were adapting to manage the increase in lipid uptake associated with the clearance of myelin debris in lesions.

Examination of DEGs further revealed higher expression of a number of genes in PPAR-δ-deficient microglia that could go further in explaining the pro-inflammatory and neurotoxic activities of these cells. Upregulated genes include *Alox5*, which encodes an enzyme that is involved in the generation of pro-inflammatory lipid mediators in MS and EAE (52), genes involved in positive regulation of toll-like receptor and IL-1-receptor signaling pathways (*Suz12, Lbp, Tab2, Card9*), monocyte/T cell clustering (53) (*Atrn*), and genes in pathways previously implicated to be pro-inflammatory in microglia (*Wnt, Camp, Spn*) (54–56) (Supplementary Table 2). Beyond *C3* and *Axl*, other genes known to be upregulated in microglia in MS lesions were also elevated with PPARδ-deficiency including *Mmp9* (57), *Cxcr2* (58). Examination of significantly downregulated pathways in microglia also revealed lowered expression of FGF receptor signaling (GO: 0008543), a pathway that is neuroprotective against glutamate-mediated excitotoxicity (59) (Supplementary Table 5) and specific genes in anti-inflammatory pathways including *Grip1* a co-activator of the glucocorticoid receptor, *Prkag3*, regulatory subunit of AMPK, and *P2ry1*, a repressor of reactive gliosis (60) (Supplementary Table 3).

Altogether, these findings highlight that in the context of EAE, PPARδ-deficiency results in microglia adopting a more reactive, neurotoxic, and potentially pro-inflammatory phenotype that is punctuated by increased expression of genes involved in ROS generation, oxidative stress, phagocytosis, and lipid clearance.

## Discussion

Past studies identified PPAR-δ as a key regulator of M2-programming, a repressor of the LPS-induction of IL-12p40, IL-6, and iNOS in macrophages (16, 38, 39, 61), and a potential regulator of human microglia activation *in vitro* (62). However, no one had yet addressed whether PPAR-δ regulates microglial phenotype *in vivo*. Here, we used a *Cx3cr1*^CreERT2^ transgene and a Cre-lox approach to induce *Ppard-*deficiency in the microglial compartment in mice. Although the *Cx3cr1*^CreERT2^ transgene is expressed in a number of myeloid populations in response to TAM treatment, by 30 days post-TAM, *Cx3cr1*^CreERT2^ expression becomes enriched in microglia. We observed that upon induction of EAE, *Cx3cr1*^CreERT2^: *Ppard*^fl/fl^ mice developed a more severe course of disease compared to controls characterized by increased microglia/monocyte activation, axonal injury, and atrophy of spinal cord white matter and certain brain regions. Although *in vitro* studies of *Ppard*-deficient microglia revealed an increase in the LPS-induction of IL-6 and IL-12p40, *Ppard*-deficient microglia did not exhibit an M1 phenotype in the context of EAE. Instead, *Ppard*-deficient microglia were more likely to aggregate in inflamed regions and retract their processes compared to control microglia. Transcriptional profiling revealed that *Ppard*-deficient microglia also exhibited lowered expression of genes associated with a homeostatic microglia phenotype, mitophagy, and FGF signaling and heightened expression of certain genes associated with NADPH production, oxidative stress, pro-inflammation, phagocytosis and lipid clearance, and M2-activation. Collectively, our findings suggest a role for PPAR-δ in microglia in limiting tissue damage during neuroinflammation.

The main finding in this study was that *Cx3cr1*^CreERT2^*:Ppard*^fl/fl^ mice exhibited more severe neuronal injury and white matter loss (−19% by 60 d) in the spinal cord in the post-acute phase of EAE as compared to controls. A similar degree of atrophy of the spinal cord has been reported previously in EAE, but not until 100 days of disease (63), suggesting tissue loss was accelerated in *Cx3cr1*^CreERT2^*:Ppard*^fl/fl^ mice. Although the clinical phenotype was most evident in males, the histological phenotype of increased myeloid inflammation and axonal injury in *Cx3cr1*^CreERT2^*:Ppard*^fl/fl^ mice was observed in both sexes, indicating that the phenotype was not sex-dependent.

The finding that microglia were the predominant Cre-expressing cell in *Cx3cr1*^CreERT2^*:Ppard*^fl/fl^ mice at 30 d post-TAM treatment coupled with the finding of an increase in neurotoxic and pro-inflammatory gene expression in microglia in *Cx3cr1*^CreERT2^*:Ppard*^fl/fl^ mice in EAE led us to conclude that *Ppard*-deficiency in microglia was the instigator of the enhanced neuronal injury in *Cx3cr1*^CreERT2^*:Ppard*^fl/fl^ mice. Of note, we did detect that a small percentage of non-microglia cells (mainly splenic DCs and monocytes) were also Cre^+^. Though examination of MOG p35-55-specific T cell responses and immune cell infiltration in the spinal cord in early EAE ruled out a role for DCs or alterations in myeloid cell trafficking as a driver of this phenotype, it remains possible that Cre^+^ (*Ppard*-deficient) monocytes also contributed to neuronal damage after maturing in the inflamed environment of the CNS.

These findings of a role for endogenous PPAR-δ in neuroprotection does correspond with past reports of neuroprotective effects of synthetic PPAR-δ agonists GW0742 and GW501516 in cultured neurons (64, 65) and in murine models of Alzheimer's disease (66, 67), stroke (68–71), Parkinson's disease (72, 73), and Huntington's disease (65). In a stroke model, activating PPAR-δ limited infarct size in the CNS by mitigating oxygen-glucose-deprivation-induced death in the vascular endothelial cells (71). Direct effects of PPAR-δ agonists on neuronal survival have been also noted in some (64, 65), but not all studies (66). For example, the PPAR-δ agonist KD3010 was shown to preserve neurons in a primary neuron culture model of Huntington's disease by restoring oxidative metabolism and enhancing autophagy (65). In contrast, another study noted no protective effect of the PPAR-δ agonist GW0742 against glutamate-induced neuron death (66); however, GW0742 did protect primary neurons from inflammation-induced neuronal death when these cells were in co-culture with M1-activated primary microglia (66). The disparity in the effects of PPAR-δ agonists in neuronal cultures, likely relates to differences in the agonists used, the doses administered, and the experimental systems utilized. Nonetheless, when considered together, these studies point toward neuroprotective effects of PPAR-δ-agonists, with effects possibly mediated through a number of cell types.

Though our data suggested a role for PPAR-δ in microglia in neuroprotection in EAE, we did not elucidate how PPAR-δ activity was mitigating neurodegeneration; however, comparison of the histological and gene expression profile of *Cx3cr1*^CreERT2^*:Ppard*^fl/fl^ and *Ppard*^fl/fl^ mice did provide us with a number of potential mechanisms. Although there was no difference in immune cell infiltration between groups early in EAE, with time, *Cx3cr1*^CreERT2^*:Ppard*^fl/fl^ mice developed enhanced monocyte/microglial inflammation as detected by increased total Iba-1 immunoreactivity. Past studies have demonstrated that both microglia and monocytes mediate focal axonal degeneration in EAE and MS lesions in EAE and MS by producing ROS, reactive nitrogen species, and expression of NADPH oxidase (14, 74, 75). In this regard, we detected higher expression of genes encoding components of the NADPH oxidase: *Nox1, Cyba, and Cybb* and a gene signature consistent with increased oxidative stress in PPAR-δ-deficient microglia. NADPH oxidase and ROS generation is known to occur as part of the respiratory burst that accompanies microglia phagocytosis of myelin debris in response to damage-associated signals encountered in lesions (76). Further, higher Nox1 NADPH oxidase activity and superoxide production has been shown to potentiate NO and IL-1 production by microglia leading to the formation of peroxynitrite, which is also neurotoxic for oligodendrocytes and neurons (77). Therefore, the increased accumulation of activated monocytes/microglia coupled with higher ROS or reactive nitrogen species generation could have been one contributor to the increased axonal injury in TAM-treated *Cx3cr1*^CreERT2^*:Ppard*^fl/fl^ mice. Alternatively, the increased axon injury in *Cx3cr1*^CreERT2^*:Ppard*^fl/fl^ mice could relate to reduced FGF receptor signaling, which is a neuroprotective pathway (59) that we observed to be downregulated in microglia from these mice.

Our results also did not pinpoint why CNS inflammation escalated to a greater extent in *Cx3cr1*^CreERT2^*:Ppard*^fl/fl^ compared to *Ppard*^fl/fl^ mice during EAE. Though our studies of cultured microglia revealed higher expression of T cell chemoattractants (IP-10 and RANTES) with PPAR-δ-deficiency, transcripts encoding these chemokines were not differentially expressed between *Cx3cr1*^CreERT2^*:Ppard*^fl/fl^ and *Ppard*^fl/fl^ microglia during EAE. On the other hand, we did detect higher expression of the gene encoding attractin (*Atrn*) in PPAR-δ-deficient microglia, which plays a role in promoting the adherence and spreading of monocytes and monocyte/T cell clustering (53) as well as higher expression of *Mmp9*, which has known disruptive effects on the blood brain barrier in EAE (57). Escalated inflammation in *Cx3cr1*^CreERT2^*:Ppard*^fl/fl^ mice could have been also promoted by an imbalance in pro-inflammatory (*Suz12, Lbp, Tab2, Traf6, Card9, Wnt3, Camp, Spn*) vs. anti-inflammatory (*Grip1, Prkag3, P2ry1*) genes in microglia, or, alternatively, to a reduction in mitophagy. Defective mitophagy in microglia increases IL-1 production in the CNS by activation of the NLRP3 inflammasome (51). It is also possible that the escalation in inflammation just occurred as a secondary consequence of the increased myelin and neuronal damage in the CNS of *Cx3cr1*^CreERT2^*:Ppard*^fl/fl^ mice. This could be better revealed through studies that examine the timing of development of neuronal injury vs. inflammation in *Cx3cr1*^CreERT2^*:Ppard*^fl/fl^ and *Ppard*^fl/fl^ mice during EAE. Altogether, the phenotype of microglia with PPAR-δ-deficiency is complex and requires further investigation to pinpoint what aspects of altered microglia function are driving inflammation vs. neurodegeneration.

Past studies that conducted bulk transcriptomic analysis and single cell RNA sequencing analysis concluded that microglia do not acquire an M1 phenotype during EAE (11, 78); instead, these cells downregulate the expression of genes associated with a homeostatic phenotype, adopt a more amoeboid appearance, and exhibit upregulated expression of a constellation of genes encoding pro-inflammatory genes (*Tnf, Ccl2 Cxcl10, Itgax, CD74*), M2-associated genes (*Chil3, Arg1, Timp2*), genes involved in phagocytosis and lipid uptake (*Axl, Apoe*), ROS production (*Cybb*), and antigen presentation (*Ly86*) (11, 78). This signature has been termed a “DAM” signature and single-cell RNA sequencing analysis demonstrated that at least 4 distinct DAM subsets emerge during EAE (78). Our comparison of *Cx3cr1*^CreERT2^*:Ppard*^fl/fl^ and *Ppard*^fl/fl^ microglia by transcriptional profiling revealed that *Cx3cr1*^CreERT2^*:Ppard*^fl/fl^ microglia had decreased expression of a number of genes associated with a homeostatic microglia signature during EAE. In addition, these cells exhibited higher expression of specific genes associated with a DAM/M2 phenotype including *Cybb* and *Timp2*, and increased expression of *Axl*, a TAM receptor family tyrosine kinase that functions in the clearance of apoptotic cells in activated microglia (46). These gene changes are not expected to have contributed to neuron damage, but instead convey that *Cx3cr1*^CreERT2^*:Ppard*^fl/fl^ microglia were adapting to deal with the increased clearance of tissue debris associated with myelin and neuronal damage.

One limitation of our study is that, in conducting bulk RNA sequencing of FACS-sorted microglia, we evaluated the transcriptome of all microglia in the spinal cord and cerebellum, which originated from lesion and extra-lesional areas. This likely resulted in a dilution of genes of interest. Our RNA sequencing study was also not well-powered to detect subtle differences in gene expression between the groups. Certainly, the application of single cell RNA sequencing would be a powerful approach to reveal how PPAR-δ-deficiency alters the composition of microglia populations in during EAE and associated gene expression within these populations (78).

In conclusion, this study revealed a role for endogenous PPAR-δ activity in limiting the neurotoxic activities of microglia in the context of CNS inflammation making this an attractive target for future therapeutic intervention in neurodegenerative disease.

## Data Availability Statement

The RNA sequencing data has been uploaded to the GEO – GSE164702.

## Ethics Statement

The animal study was reviewed and approved by University Health Network Animal Care Committee and St. Michael's Hospital Animal Care Committee.

## Author's Note

MS is an autoimmune disease that targets the myelin sheath of central nervous system neurons resulting in the formation of inflammatory demyelinating lesions and focal axonal injury. The production of reactive oxygen and nitrogen species by microglia and monocytes is considered to be the cause of this axonal injury. With time the injury elicited by these attacks leads to permanent neuron loss and the accrual of disability. Although there exist a number of treatments to prevent autoimmune attacks in MS, there are very few treatments available that can protect neurons from damage. Here we describe a neuroprotective role for the nuclear receptor PPAR-δ in microglia in the animal model of MS called EAE. We observed that mice deficient in this molecule in microglia exhibited an increased expression of genes that describe a DAM signature including transcripts encoding the NADPH oxidase, a major producer of intracellular reactive oxygen species, as well as more extensive axonal injury during EAE. PPAR-δ may therefore be a molecular target for therapy in protecting neurons from damage during neuroinflammation.

## Author Contributions

SD conceived the study. ED, PD, and SD conducted EAE experiments and wrote the manuscript. AG, HW, and MG conducted IF, IHC, and Sholl analysis. LC, SS, and JS conducted MRI analysis. TY maintained mice and helped with flow cytometry. All authors have approved the manuscript for publication.

## Conflict of Interest

The authors declare that the research was conducted in the absence of any commercial or financial relationships that could be construed as a potential conflict of interest.
