# Supplementary material for: Peroxisome Proliferator-Activated Receptor-δ Deficiency in Microglia Results in Exacerbated Axonal Injury and Tissue Loss in Experimental Autoimmune Encephalomyelitis
Source: Front Immunol. 2021 Feb 26;12:570425. doi: 10.3389/fimmu.2021.570425 (PMC7959796; doi:10.3389/fimmu.2021.570425)
Supplement: Supplementary file 1 [file Data_Sheet_1.docx]

Supplementary Material

# Supplementary Data

## Supplementary Figures

**Supplementary Figure 1.** ***Ppard*^-/-^ *m*icroglia grow more slowly and produce higher levels of pro-inflammatory mediators compared to *Ppard*^+/+^ microglia *in vitro*.** **(A-F)** *Ppard*^+/+^ and *Ppard*^-/-^ microglia grown with M-CSF were plated at 0.1 x 10^6^/well in media containing M-CSF. Proliferation was measured by flow cytometry using either BrdU incorporation or CFSE dilution assays. **(A)** Representative flow cytometry staining and gating of microglia for evaluation of proliferation. **(B)** Number of *Ppard*^-/-^ microglia recovered from cultures, expressed as a mean + SEM percentage of *Ppard*^+/+^ numbers. Values are a mean + SEM of two independent experiments. **(C)** Frequency of BrdU^+^ microglia at 24 and 48 h (peak of incorporation). **(D)** Frequency of microglia that divided at 48 and 72 h. **(E)** Shows the frequency of viability dye negative cells in singlet gate at 48 and 72 h. **(F)** Percentage of CD45^lo^CD11b^+^ cells in the live singlet gate at each time point. Graphs in C-F are representative of three individual experiments (one in male and two in female) and show the mean + SEM values of individual wells obtained in one experiment. **(G-N)** Microglia from *Ppard^+/+^* and *Ppard^-/-^* mice were grown in culture and then were re-plated at a specific density overnight with 150 U/ml IFN-γ followed by stimulation the following day with 10 ng/ml LPS. Levels of the chemokine CXCL10 **(G),** prostaglandin E_2_ **(H)** were measured by ELISA at 24 h. Cytokines were evaluated in culture supernatants by ELISA at 6 h (IL-6) or after 24 h (other cytokines). Shown are the levels of IL-6 **(I)**, TNFα **(J)**, RANTES **(K)**, nitrite levels measured by Griess assay **(L)**, IL-12p40 **(M)**, and IL-10 **(N)**. Values are mean + SEM of triplicate wells in one experiment and are representative of three independent experiments (2 done in females and 1 done in males). * Significantly different from *Ppard*^+/+^ counterpart (P < 0.05) by two-tailed t-test (two-tailed).

#

**Supplementary Figure 2.** ***Cx3cr1*^CreERT2^ transgene recombination is highly efficient in microglia at 30-60 days following TAM treatment.** Mice that were homozygous for the *Cx3cr1*^CreERT2^ transgene were crossed to a Cre-reporter strain that have a cassette that encodes a flox-stop-flox-td-Tomato transgene targeted to the Rosa26 locus (*R26-td-Tomato mice*). *Cx3cr1*^CreERT2^:*R26-td-Tomato*, *R26-td-Tomato* mice (6 weeks of age, n = 3-5 per group) were treated to two 10 mg doses of tamoxifen (TAM) by oral gavage, separated by 48 h. Mice were sacrificed at day 30 following the first TAM dose to assess td-Tomato expression in the various cell populations. (A) Representative staining of td-Tomato in microglia (eYFP^+^CD45^lo^CD11b^+^), CNS CD45^hi^CD11b^+^ cells, spleen dendritic cells (CD45^+^CD11b^int^), spleen monocyte/macrophages (CD45^+^SSC^lo^CD11b^+^), granulocytes (CD45^+^SSC^hi^CD11b^+^), and non-CD11b cells. These cells had been pre-gated on singlets and live cells. (B) Mean + SEM percent frequency of td-Tomato^+^ cells for each subset. Data are representative of two independent experiments that were performed. (C) RT-PCR of *Ppard* mRNA expression in of *Cx3cr1*^CreERT2^:*Ppard*^fl/fl^ and *Ppard*^fl/fl^ FACS-sorted microglia (n=4 mice per group) following more than 60 days of TAM administration, dosed as previously described.* different from *Ppard*^fl/fl^ by Mann-Whitney test (P<0.05).

**Supplementary Figure 3. Two injections of TAM can inhibit myelin-specific Th17 responses and inhibit the accumulation of immune cells in mice even at 30 days post-injection.**

(A-C) C57BL6/J mice were provided two injections of tamoxifen (TAM) or vehicle. Thirty days later, these mice were immunized with MOG p35-55/CFA and the recall responses to MOG p35-55 were examined in the spleen at 9 days post-immunization. (A) Proliferation of spleen cells to

MOG p35-55 between 24-42 h by [^3^H]-thymidine incorporation assay. CPM=counts per minute.

(B-C) IFN-γ and IL-17A levels in in culture supernatants as determined by ELISA

assay. *Different from vehicle by two-tailed T test (P<0.05). Results in A-C are representative of 3 studies.

(D) *Cx3cr1*^CreERT2^: *Ppard*^fl/fl^ mice were injected twice with tamoxifen or vehicle and then EAE was induced 30 days later with MOG p35-55/CFA and pertussis toxin. At 3 days post onset of symptoms, CNS mononuclear cells were isolated by Percoll gradient and were counted. Results in D are from an experiment that was performed in parallel to the experiment shown in Fig. 1C. **Different by two-tailed T test (P<0.05).


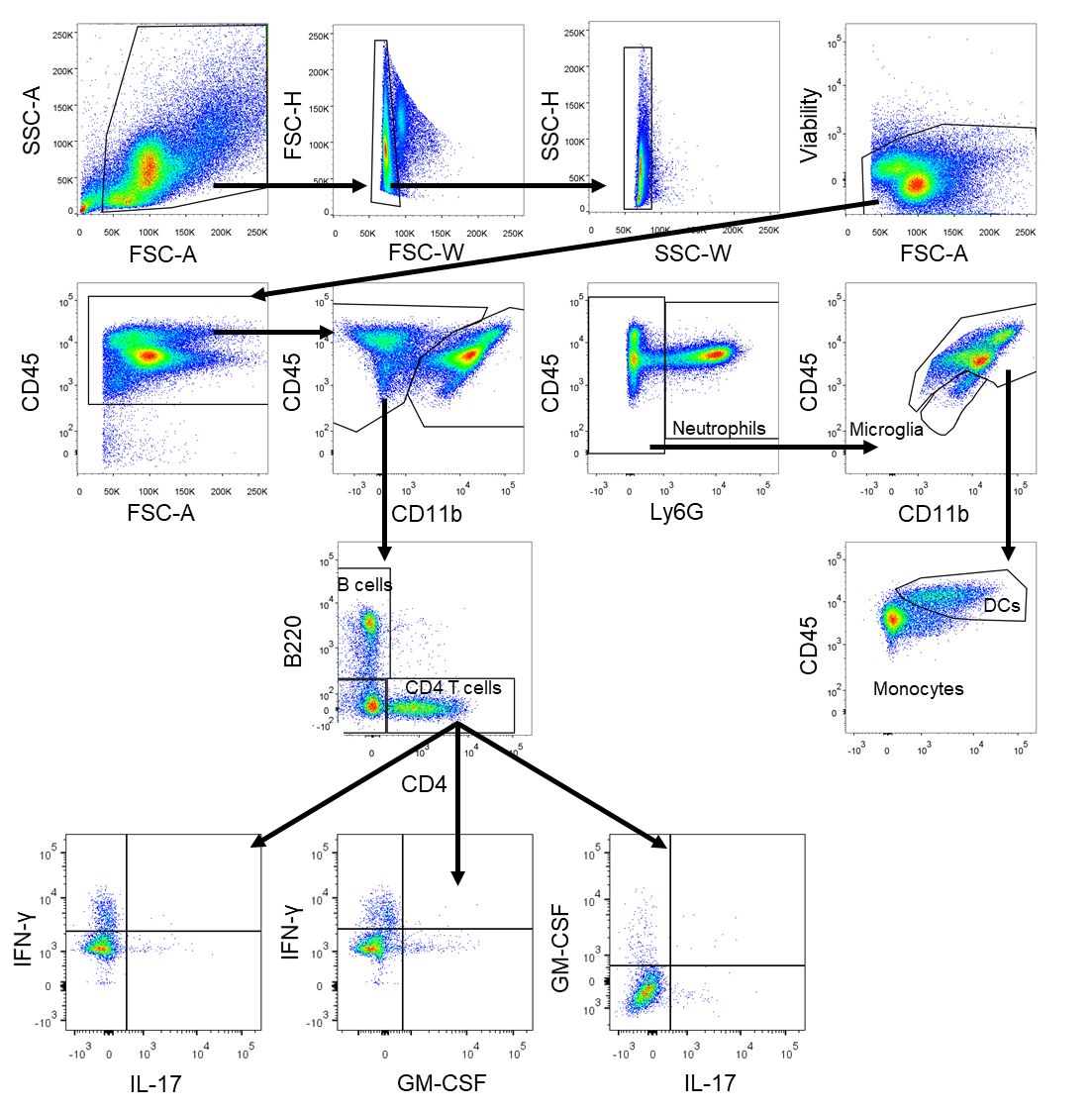


**Supplementary Figure 4. CNS mononuclear flow gating strategy.** Mononuclear cells isolated from the CNS of TAM-treated *Cx3cr1*^CreERT2^: *Ppard*^fl/fl^ and *Ppard*^fl/fl^ mice at the peak of EAE. Cells were stained for flow cytometry, and then gated for singlets and live lymphocytes and then by the expression of CD45 to identify leukocytes. Subsequently cells were gated to identify neutrophils (CD45^+^CD11b^+^Ly6G^+^), microglia (CD45^lo^CD11b^+^Ly6G^-^), monocyte/macrophages (CD45^hi^CD11b^+^Ly6G^-^CD11c^-^), DCs (CD45^hi^CD11b^+^Ly6G^-^CD11c^+^), B cells (CD45^+^CD11b^-^B220^+^), and CD4 T cells (CD45^+^CD11b^-^CD4^+^). On samples stimulated with PMA/ionomycin, CD4 T cell cytokine response was evaluated with intracellular staining, as is shown in representative plots in the bottom row of the panel.

*Ppard*^fl/fl^ *Cx3cr1*^CreERT2^: *Ppard*^fl/fl^

**Supplementary Figure 5. Microglia are more reactive in certain brain regions of *Cx3cr1*^CreERT2^: *Ppard*^fl/fl^ mice during EAE.**

*Cx3cr1*^CreERT2^: *Ppard*^fl/fl^  and *Ppard*^fl/fl^ were injected twice with tamoxifen, two days apart and then after 30 days were induced with EAE by immunization with MOG p35-55/CFA and pertussis toxin. After 45 days, mice were killed for histological analysis of Iba-1 expression in the brain. Shown are microglia in stained with Iba-1 antibody by DAB immunohistochemistry **(A-H)**. Representative Iba-1 stained areas in the indicated brain areas as visualized by DAB substrate from *Ppard*^fl/fl^ **(A, C, E, G)** and *Cx3cr1*^CreERT2^*:Ppard*^fl/fl^ **(B, D, F, H)** mice. **(A-D,G,H)** Scale bar = 20µm, **(E, F)** Scale bar = 5 µm.

**Supplementary Figure 6. Representative Iba-1/ TMEM119/DAPI staining in the brain and spinal cord.** (**A-D**) Show a scanned image of microglia in the spinal cord white matter co-stained with TMEM119 (**A**), Iba-1 (**B**), DAPI (inset DAPI plus 2ndary antibodies) (**C**), and the merge of all three labels (**D**). Scale bar= 50 µm. (**E-G**) Show representative microglia in *Ppard*^fl/fl^ and *Cx3cr1*^CreERT^*:Ppard*^fl/fl^ (F, G) mice. Scale bar is 20 µm. In E, the arrow points to a damaged axon stained non-specifically for TMEM119 antibody. We observed that TMEM119 antibody does label microglia, but also lightly labels neuron cell bodies in the grey matter (not shown) and some injured neurons.

## Supplementary Tables

## Supplementary Table 1. Volumes of different brain regions in TAM and vehicle (corn-oil)-treated *Cx3cr1*^CreERT2^: *Ppard*^fl/fl^ mice at 58 days post-onset of EAE

|  | **Naïve C57BL6/J** | | ***Cx3cr1*^CreER^*:Ppard*^fl/fl^**  **(corn oil)** | | ***Cx3cr1*^CreER^*:Ppard*^fl/fl^**  **(tamoxifen)** | |  |
| --- | --- | --- | --- | --- | --- | --- | --- |
|  | **Mean** | **SEM** | **Mean** | **SEM** | **Mean** | **SEM** | **Uncorrected**  **P value** |
| **fasciculus retroflexus** | 0.267776 | 0.005609 | 0.249035 | 0.005989 | 0.244192 | 0.002889 | 0.0013* |
| **bed nucleus of stria terminalis** | 1.512661 | 0.020000 | 1.4369 | 0.0711 | 1.359872 | 0.024582 | 0.0018* |
| **lateral septum** | 3.3858 | 0.0243 | 3.222869 | 0.105267 | 3.110933 | 0.055867 | 0.0034* |
| **stria medullaris** | 0.721109 | 0.019314 | 0.671381 | 0.014127 | 0.67 | 0.01 | 0.0036* |
| **medial septum** | 1.298880 | 0.008868 | 1.238272 | 0.031944 | 1.163605 | 0.020040 | 0.0037* |
| **anterior commissure: pars posterior** | 0.492928 | 0.003950 | 0.462443 | 0.019382 | 0.446133 | 0.009184 | 0.0040* |
| **internal capsule** | 2.8674 | 0.0721 | 2.7155 | 0.0909 | 2.656661 | 0.030129 | 0.0042* |
| **striatum** | 22.735317 | 0.209194 | 21.07641 | 0.76807 | 20.7731 | 0.4299 | 0.0049* |
| **nucleus accumbens** | 4.488555 | 0.038167 | 4.205152 | 0.150063 | 4.09612 | 0.07622 | 0.0056* |
| **stratum granulosum of hippocampus** | 0.937259 | 0.011923 | 0.88126 | 0.01625 | 0.808405 | 0.024733 | 0.0060* |
| **globus pallidus** | 3.289259 | 0.091610 | 3.165227 | 0.169239 | 3.048853 | 0.040435 | 0.0070* |
| **fimbria** | 3.534592 | 0.061362 | 3.385707 | 0.107937 | 3.35247 | 0.03187 | 0.010* |
| **anterior commissure: pars anterior** | 1.528107 | 0.060306 | 1.460405 | 0.055109 | 1.386261 | 0.021480 | 0.011* |
| **dentate gyrus of hippocampus** | 3.627627 | 0.038097 | 3.476501 | 0.055241 | 3.267701 | 0.083105 | 0.012* |
| **optic tract** | 1.6309 | 0.0396 | 1.523168 | 0.051244 | 1.501995 | 0.021289 | 0.021* |
| **stria terminalis** | 0.959275 | 0.021775 | 0.904096 | 0.030018 | 0.903531 | 0.012074 | 0.025* |
| **amygdala** | 14.752043 | 0.290764 | 14.554709 | 0.736344 | 13.982357 | 0.145512 | 0.029 |
| **mammilothalamic tract** | 0.2459 | 0.0055 | 0.234880 | 0.005446 | 0.233504 | 0.002953 | 0.031 |
| **cerebral peduncle** | 2.251285 | 0.079041 | 2.231157 | 0.054547 | 2.09082 | 0.03888 | 0.034 |
| **fundus of striatum** | 0.183979 | 0.007303 | 0.16789 | 0.00999 | 0.161952 | 0.004373 | 0.037 |
| **basal forebrain** | 5.518891 | 0.064090 | 5.37679 | 0.21478 | 5.165408 | 0.090639 | 0.038 |
| **olfactory bulbs** | 26.995840 | 0.437367 | 25.38892 | 0.54385 | 25.713376 | 0.387439 | 0.042 |
| **lateral olfactory tract** | 1.279829 | 0.035791 | 1.250741 | 0.029961 | 1.218731 | 0.012211 | 0.048 |
| **fornix** | 0.675328 | 0.014317 | 0.651264 | 0.021114 | 0.64137 | 0.01003 | 0.050 |
| **olfactory tubercle** | 3.474880 | 0.048150 | 3.325035 | 0.039950 | 3.095691 | 0.060650 | 0.056 |
| **pre-para subiculum** | 2.098645 | 0.035180 | 2.0053 | 0.0421 | 1.97927 | 0.04091 | 0.060 |
| **hippocampus** | 19.666453 | 0.288583 | 20.163563 | 0.748583 | 18.935371 | 0.235353 | 0.065 |
| **cerebral aqueduct** | 0.400021 | 0.013768 | 0.440533 | 0.017441 | 0.428491 | 0.009572 | 0.068 |
| **hypothalamus** | 10.87051 | 0.20780 | 10.772 | 0.306 | 10.4497 | 0.1101 | 0.072 |
| **corpus callosum** | 16.561835 | 0.514703 | 15.876043 | 0.594169 | 15.58924 | 0.36989 | 0.083 |
| **cuneate nucleus** | 0.206080 | 0.008184 | 0.235285 | 0.006956 | 0.233824 | 0.007828 | 0.10 |
| **subependymale zone / rhinocele** | 0.068885 | 0.001526 | 0.06818 | 0.00252 | 0.066240 | 0.001330 | 0.13 |
| **cerebral cortex: frontal lobe** | 40.877184 | 0.529989 | 40.848608 | 1.150129 | 39.33251 | 0.76103 | 0.14 |
| **thalamus** | 17.299797 | 0.298900 | 17.030165 | 0.401007 | 16.824971 | 0.188888 | 0.15 |
| **interpedunclar nucleus** | 0.2526 | 0.0056 | 0.260704 | 0.004957 | 0.268096 | 0.004379 | 0.15 |
| **colliculus: superior** | 8.738581 | 0.101836 | 8.296416 | 0.243878 | 8.354517 | 0.145207 | 0.16 |
| **cerebral cortex: parieto-temporal lobe** | 74.570325 | 0.702301 | 73.390795 | 1.933389 | 71.876853 | 1.241212 | 0.16 |
| **medulla** | 24.985195 | 0.544505 | 26.559829 | 0.423018 | 25.892309 | 0.389431 | 0.17 |
| **fourth ventricle** | 0.36250 | 0.00808 | 0.379449 | 0.017720 | 0.372939 | 0.006588 | 0.22 |
| **cerebellar cortex** | 41.144960 | 0.917088 | 41.664864 | 0.610019 | 42.18141 | 0.67957 | 0.25 |
| **cerebral cortex: occipital lobe** | 5.584384 | 0.093501 | 5.638336 | 0.082868 | 5.326827 | 0.145170 | 0.25 |
| **facial nerve (cranial nerve 7)** | 0.22707 | 0.00445 | 0.22370 | 0.00516 | 0.221227 | 0.002878 | 0.25 |
| **ventral tegmental decussation** | 0.123755 | 0.001334 | 0.127435 | 0.004399 | 0.126368 | 0.001730 | 0.31 |
| **corticospinal tract/pyramids** | 1.489387 | 0.059566 | 1.480181 | 0.051367 | 1.57091 | 0.02882 | 0.32 |
| **cerebral cortex: entorhinal cortex** | 10.148437 | 0.055045 | 10.311445 | 0.215664 | 9.899083 | 0.182102 | 0.33 |
| **pons** | 16.918464 | 0.356339 | 16.975595 | 0.276700 | 16.571957 | 0.163593 | 0.36 |
| **posterior commissure** | 0.134315 | 0.001944 | 0.13377 | 0.00336 | 0.136107 | 0.001563 | 0.36 |
| **medial lemniscus/medial longitudinal fasciculus** | 2.432085 | 0.052159 | 2.456544 | 0.056428 | 2.523605 | 0.052462 | 0.49 |
| **midbrain** | 14.094656 | 0.152483 | 13.720597 | 0.387328 | 13.924224 | 0.160808 | 0.52 |
| **habenular commissure** | 0.023253 | 0.001355 | 0.02522 | 0.00243 | 0.022240 | 0.001739 | 0.53 |
| **periaqueductal grey** | 4.005867 | 0.055662 | 3.9020 | 0.0778 | 3.940181 | 0.044525 | 0.65 |
| **cerebellar peduncle: inferior** | 0.759829 | 0.015442 | 0.792939 | 0.018420 | 0.759413 | 0.007833 | 0.66 |
| **mammillary bodies** | 0.419029 | 0.015296 | 0.433909 | 0.015110 | 0.417909 | 0.008709 | 0.67 |
| **arbor vita of cerebellum** | 9.990315 | 0.325759 | 9.551637 | 0.224902 | 9.784821 | 0.173818 | 0.70 |
| **inferior olivary complex** | 0.282304 | 0.010328 | 0.290848 | 0.012328 | 0.28621 | 0.01384 | 0.78 |
| **third ventricle** | 1.142187 | 0.052557 | 1.21051 | 0.03176 | 1.15294 | 0.02442 | 0.82 |
| **colliculus: inferior** | 5.655445 | 0.167076 | 5.44235 | 0.10074 | 5.70064 | 0.10345 | 0.85 |
| **superior olivary complex** | 0.63138 | 0.01695 | 0.660192 | 0.014585 | 0.629355 | 0.022730 | 0.86 |
| **cerebellar peduncle: superior** | 1.031147 | 0.014468 | 1.025611 | 0.025931 | 1.025451 | 0.013409 | 0.88 |
| **pontine nucleus** | 0.637739 | 0.054651 | 0.6940 | 0.0207 | 0.640213 | 0.021134 | 0.88 |
| **cerebellar peduncle: middle** | 1.11844 | 0.05810 | 1.162688 | 0.021494 | 1.100821 | 0.025057 | 0.90 |
| **lateral ventricle** | 3.870123 | 0.308998 | 3.524949 | 0.138155 | 3.864811 | 0.176236 | 0.96 |

Values representative of volumes in mm^3^. The p values are marked with an asterisk (*) if the difference between *Cx3cr1*^CreERT2^*:Ppard*^fl/fl^ (tamoxifen) and B6 mice was significant at an FDR of 10%. B6, n = 3 mice; *Cx3cr1*^CreERT2^*:Ppard*^fl/fl^ (corn oil), n = 6 mice; *Cx3cr1*^CreERT2^*:Ppard*^fl/fl^ (tamoxifen), n = 8 mice. Data are from one independent experiment.

**Supplementary Table 2: Genes found to be significantly upregulated in *Cx3cr1*^CreERT2^: *Ppard*^fl/fl^ versus *Ppard*^fl/fl^ microglia**

| **gene_name** | **Sample 1**  ***Cx3cr1*^CreERT2^: *Ppard*^fl/fl^** | **Sample 2 *Cx3cr1*^CreERT2^: *Ppard*^fl/fl^** | **Sample 1**  ***Ppard*^fl/fl^** | **Sample 2**  ***Ppard*^fl/fl^** | **P-Value** | **Fold-change** |
| --- | --- | --- | --- | --- | --- | --- |
| *Ppm1g* | 4.822712105 | 4.838870529 | 2.948732182 | 2.965990765 | 3.98121E-05 | 1.633480168 |
| *Fstl3* | 15.51227893 | 15.54311276 | 9.678814 | 9.778981164 | 8.16542E-05 | 1.596038576 |
| *Pdlim7* | 0.411970068 | 0.406007454 | 0 | 0 | 5.3132E-05 |  |
| *Abhd11* | 2.750785051 | 2.700672715 | 1.382021021 | 1.367428218 | 0.000372925 | 1.982745376 |
| *Emc8* | 2.513429031 | 2.477170578 | 1.578962788 | 1.5568235 | 0.000524195 | 1.591498639 |
| *Plxnb2* | 1.127228001 | 1.116593229 | 0.939417725 | 0.938587075 | 0.000849216 | 1.194789933 |
| *Klhl18* | 4.336860192 | 4.282088824 | 1.390850811 | 1.547674402 | 0.000854062 | 2.933086631 |
| *Mon2* | 0.530827765 | 0.508551951 | 0.123444179 | 0.131893454 | 0.000922071 | 4.070609193 |
| *Wdr43* | 2.042771 | 2.055434 | 0.469933 | 0.356812 | 0.001208436 | 4.957036329 |
| *Csgalnact2* | 4.659088991 | 4.536141197 | 2.993781006 | 3.054281381 | 0.001890345 | 1.520359679 |
| *Gcn1l1* | 0.853882963 | 0.881044187 | 0.350635934 | 0.386115324 | 0.00199782 | 2.354834323 |
| *Lrwd1* | 6.862286133 | 6.643808711 | 4.102119649 | 4.210085559 | 0.002194267 | 1.624850988 |
| *Eif3l* | 3.244049 | 3.565913 | 0 | 0 | 0.0022264 |  |
| *Marchf8* | 3.239823826 | 3.097473293 | 0.266029077 | 0.507729553 | 0.00253236 | 8.190276499 |
| *Nudt3* | 11.97516779 | 11.56043587 | 6.257923169 | 6.641220632 | 0.002807083 | 1.824586501 |
| *Zdhhc5* | 4.443469165 | 4.584714436 | 3.142464835 | 3.095029063 | 0.002838475 | 1.4474056 |
| *Atxn7* | 0.744522 | 0.738433 | 0.167936 | 0.233453 | 0.003680731 | 3.694558147 |
| *Zfyve27* | 0.958941849 | 0.999921232 | 0.630617621 | 0.642530007 | 0.003850871 | 1.538598539 |
| *Stxbp5* | 0.65646248 | 0.726110112 | 0.091859947 | 0.114596098 | 0.003858074 | 6.696692239 |
| *Ccdc175* | 2.5797 | 2.472715 | 1.031568 | 0.850733 | 0.004364174 | 2.684169535 |
| *Dnm1l* | 1.489149317 | 1.356912047 | 0.322004932 | 0.384815763 | 0.004650541 | 4.026567676 |
| *Alas1* | 17.55057648 | 18.53714504 | 6.8081114 | 7.895938436 | 0.004683417 | 2.454270893 |
| *Mttp* | 2.058565837 | 2.117575858 | 1.279586936 | 1.366795108 | 0.004704485 | 1.578056995 |
| *4932438A13Rik* | 0.550045645 | 0.539938835 | 0.029462252 | 0.096046272 | 0.004840472 | 8.684545437 |
| *Galnt7* | 9.612612913 | 9.302535883 | 5.112248245 | 5.606211956 | 0.005024676 | 1.764726317 |
| *Braf* | 1.322453598 | 1.250126599 | 0.173373424 | 0.304989151 | 0.005103211 | 5.377887675 |
| *Alox5* | 5.613766207 | 5.992108393 | 1.879704692 | 2.269012015 | 0.005257777 | 2.797461341 |
| *Akt2* | 0.372478645 | 0.415588339 | 0 | 0 | 0.002979062 |  |
| *Cyfip1* | 2.670751615 | 2.770766259 | 1.299094497 | 1.468085165 | 0.005348328 | 1.966449071 |
| *Dennd1b* | 0.314111295 | 0.344926122 | 0.100587079 | 0.083479153 | 0.00546135 | 3.580436292 |
| *Mpeg1* | 19.797277 | 20.931278 | 12.851798 | 12.772441 | 0.005616625 | 1.589454227 |
| *Mpv17l* | 0.762703676 | 0.77450267 | 0 | 0.108778806 | 0.005816343 | 14.13148752 |
| *4930430F08Rik* | 3.451091 | 3.257446 | 1.184702 | 0.872221 | 0.00618785 | 3.261442942 |
| *Aqr* | 1.100219445 | 1.127834375 | 0 | 0.175348563 | 0.007395247 | 12.70642756 |
| *Naaa* | 2.170074137 | 1.898578954 | 0.296057202 | 0.106717404 | 0.008054175 | 10.10156308 |
| *Ptgir* | 13.1538197 | 12.13057228 | 3.781845876 | 4.883301065 | 0.008084261 | 2.917941514 |
| *Cyba* | 73.458 | 81.301064 | 31.02482 | 33.377697 | 0.008112736 | 2.402997138 |
| *Bricd5* | 64.576004 | 72.274345 | 25.390085 | 23.020111 | 0.008193265 | 2.826891033 |
| *Dpcd* | 16.54975909 | 18.34653351 | 3.800471235 | 5.315921064 | 0.008210962 | 3.827862103 |
| *Spn* | 5.069000729 | 5.205974859 | 3.347492599 | 3.015473066 | 0.008324093 | 1.614809215 |
| *Ggnbp2* | 3.062547209 | 3.233528192 | 2.139959797 | 2.196423404 | 0.008337098 | 1.451918594 |
| *Tmem164* | 1.927101025 | 2.111096646 | 0.884011043 | 0.968115249 | 0.008455807 | 2.180303627 |
| *Ranbp9* | 11.203132 | 12.267413 | 5.616205 | 5.932634 | 0.008562826 | 2.032286103 |
| *Dgkq* | 4.701597331 | 4.537551768 | 2.585701816 | 2.901099037 | 0.00885715 | 1.683886357 |
| *Atrn* | 0.682068484 | 0.656961898 | 0.086356875 | 0.184935544 | 0.008955022 | 4.935745663 |
| *Wipi1* | 1.185226915 | 1.241936583 | 0.263941942 | 0.429934739 | 0.010087203 | 3.497975306 |
| *Tsc22d4* | 3.099574419 | 3.11832527 | 0.992697671 | 0.506637706 | 0.01046043 | 4.147103967 |
| *Kansl1* | 1.506723263 | 1.719573614 | 0.561385957 | 0.473379017 | 0.010866009 | 3.117903057 |
| *Gtf2a1* | 3.937461029 | 4.029437042 | 1.305164646 | 1.809965623 | 0.011000471 | 2.557484723 |
| *Apbb1ip* | 3.198950603 | 2.934795237 | 1.182221169 | 0.827796923 | 0.011296009 | 3.051587379 |
| *Guf1* | 2.342160629 | 2.438893738 | 1.511827965 | 1.662472142 | 0.012188358 | 1.506175914 |
| *Mpp1* | 2.136412755 | 2.08687889 | 0.984154548 | 0.697183856 | 0.012871867 | 2.511862952 |
| *Sh2b3* | 5.41868107 | 5.892065834 | 3.570990099 | 3.388752389 | 0.013320527 | 1.625167443 |
| *Pfdn2* | 1.62398508 | 1.97487517 | 0 | 0.195439644 | 0.013642674 | 18.41417729 |
| *Toe1* | 9.787300218 | 10.8712483 | 5.497807127 | 4.903758277 | 0.014213501 | 1.986099949 |
| *Amz1* | 9.138135379 | 9.046093401 | 7.363384152 | 6.895605914 | 0.014433192 | 1.275281678 |
| *Pced1a* | 2.621104656 | 2.742663286 | 1.611881842 | 1.343364324 | 0.014648211 | 1.814998698 |
| *Flna* | 10.50323775 | 9.742769512 | 3.238482991 | 4.576856371 | 0.014990839 | 2.590547425 |
| *Trim15* | 2.967779799 | 2.396269261 | 0.290158311 | 0.391493237 | 0.015020482 | 7.869195159 |
| *Ccnc* | 1.77533619 | 2.040485255 | 0.816470953 | 0.715669682 | 0.015080793 | 2.490516444 |
| *Nox1* | 7.526825729 | 6.357865223 | 2.265983409 | 2.35420269 | 0.015636569 | 3.005223308 |
| *Med16* | 1.068809961 | 1.33450925 | 0 | 0.118655116 | 0.015837892 | 20.25466154 |
| *Zfp740* | 2.976519072 | 3.337603036 | 1.516219945 | 1.224597985 | 0.01645628 | 2.303736428 |
| *4933428G20Rik* | 10.747714 | 12.941258 | 3.265848 | 3.505712 | 0.016591022 | 3.498303493 |
| *Dlst* | 4.056764 | 3.215 | 0.409398 | 0.411399 | 0.016603934 | 8.859393979 |
| *Dok3* | 7.93215 | 9.197797 | 1.860402 | 0.380142 | 0.016682354 | 7.645441018 |
| *Me2* | 2.349258 | 2.84943 | 0.389462 | 0 | 0.016934575 | 13.34838315 |
| *Cct6a* | 0.643073412 | 0.66082017 | 0 | 0.151058678 | 0.016964773 | 8.631702562 |
| *Add3* | 7.460325691 | 6.692606926 | 2.978067114 | 3.648683106 | 0.017855052 | 2.135727491 |
| *Lmf2* | 2.871176 | 2.596047 | 0.13411 | 0.700194 | 0.017960669 | 6.553034625 |
| *Mmp9* | 1.403062913 | 1.662010674 | 0.263856012 | 0.453056082 | 0.018146903 | 4.275382732 |
| *Pacs1* | 0.880364 | 1.129645 | 0 | 0 | 0.015034921 |  |
| *Kif11* | 2.281477 | 2.717499 | 0.917638 | 0.922355 | 0.01852561 | 2.716845118 |
| *Cbx4* | 0.774392709 | 0.746500662 | 0.541578269 | 0.477632032 | 0.018794496 | 1.492227236 |
| *Tubg1* | 4.413893234 | 3.621981829 | 1.004416192 | 1.18930171 | 0.018830112 | 3.66313055 |
| *Tial1* | 0.657309469 | 0.566504684 | 0.1653664 | 0.236925638 | 0.019235228 | 3.042103836 |
| *Tab2* | 1.34478338 | 1.615261909 | 0.155425722 | 0.367092548 | 0.019281349 | 5.664960363 |
| *Mapt* | 3.980911869 | 3.605132993 | 2.230029982 | 2.407869596 | 0.01931142 | 1.635663889 |
| *Smyd5* | 1.558136145 | 1.339000458 | 0.578360628 | 0.660450019 | 0.019336418 | 2.338643608 |
| *Wnt3* | 1.08411 | 1.408149 | 0 | 0 | 0.016487805 |  |
| *AB124611* | 9.96602837 | 10.98321103 | 0.53148678 | 2.819280859 | 0.019646169 | 6.252071661 |
| *Hp1bp3* | 0.467875203 | 0.424553036 | 0.235060787 | 0.182882382 | 0.019823944 | 2.135286101 |
| *Mxi1* | 2.428808026 | 2.153992179 | 1.329069791 | 1.335426022 | 0.019922748 | 1.719950236 |
| *Cxcr2* | 2.532649066 | 2.107157443 | 0.67937992 | 0.396054951 | 0.019954801 | 4.314353788 |
| *Hnrnpf* | 13.82910149 | 15.42032638 | 7.955380093 | 6.560806009 | 0.020007207 | 2.014952665 |
| *Tsn* | 0.548931585 | 0.462925238 | 0.191575709 | 0.14749531 | 0.020017354 | 2.984203211 |
| *Narf* | 0.491013667 | 0.656272447 | 0 | 0 | 0.020124249 |  |
| *Tspo* | 5.988201422 | 7.325036957 | 1.095140834 | 1.782735936 | 0.020128373 | 4.626062698 |
| *Tctn1* | 90.4459994 | 96.04945131 | 41.07789936 | 53.29986255 | 0.020646113 | 1.976052906 |
| *Zfyve9* | 0.934542 | 1.219967 | 0 | 0 | 0.017101502 |  |
| *Psmc3ip* | 19.29985137 | 19.08524854 | 11.34056439 | 13.3455932 | 0.020988031 | 1.55492404 |
| *Cbl* | 1.727965497 | 1.948982091 | 0.357157258 | 0.677855028 | 0.021049631 | 3.552564195 |
| *Apc* | 0.161802715 | 0.198865979 | 0.050890317 | 0.056345471 | 0.021159823 | 3.363323947 |
| *Pkig* | 2.659163066 | 2.249944499 | 1.015071992 | 0.81409574 | 0.021216477 | 2.683793005 |
| *Ap2b1* | 1.98950307 | 1.800391919 | 0.634311901 | 0.910200458 | 0.021477932 | 2.45378094 |
| *Avpr2* | 63.64654497 | 67.90516409 | 25.22047532 | 34.96891835 | 0.021507804 | 2.185629412 |
| *Trpt1* | 24.280218 | 27.779566 | 11.435448 | 13.537891 | 0.021969561 | 2.084614476 |
| *Tbcel* | 1.02575414 | 0.998236141 | 0.173333333 | 0.392616991 | 0.022212573 | 3.576268437 |
| *Atp2b2* | 0.503171475 | 0.490079096 | 0.044553221 | 0.162998214 | 0.022239065 | 4.785563499 |
| *Cdc23* | 10.41119896 | 12.08784633 | 4.391741897 | 5.35770124 | 0.022267482 | 2.307726193 |
| *Hspa14* | 0.570651163 | 0.6709666 | 0.297208875 | 0.280768155 | 0.022668108 | 2.148212989 |
| *C3* | 29.35071408 | 29.81184659 | 6.704736924 | 12.77531516 | 0.022737807 | 3.037084316 |
| *Emg1* | 9.887005156 | 8.806413488 | 5.863813841 | 5.641635582 | 0.022754624 | 1.624744758 |
| *Tead2* | 5.01777095 | 5.106939757 | 2.826046003 | 3.416330982 | 0.022837973 | 1.621931955 |
| *Mxd4* | 0.691157876 | 0.753660631 | 0 | 0.185682587 | 0.023365742 | 7.781120068 |
| *Bysl* | 147.763123 | 157.515121 | 70.094131 | 90.503716 | 0.023581892 | 1.90088628 |
| *Cox15* | 5.772256917 | 6.225306772 | 4.035671202 | 4.370490283 | 0.023732489 | 1.427234501 |
| *Kif2a* | 1.857552487 | 1.894129461 | 0.660606952 | 0.989451941 | 0.023901308 | 2.273665482 |
| *2310033P09Rik* | 16.54607 | 18.226198 | 10.815957 | 11.75104 | 0.023931392 | 1.540846042 |
| *Hbp1* | 17.33776282 | 19.97615094 | 10.13307431 | 10.40190374 | 0.024083566 | 1.81709051 |
| *Suz12* | 1.555085669 | 1.513880425 | 0.633130811 | 0.877836639 | 0.024442371 | 2.031126544 |
| *Rgs19* | 2.798748637 | 2.829297359 | 1.324568305 | 0.759048762 | 0.024593096 | 2.70109421 |
| *Nrp2* | 0.301412608 | 0.262520036 | 0.095221683 | 0.132567205 | 0.024777867 | 2.475681091 |
| *Mgat5* | 0.891780536 | 0.989344176 | 0.352449223 | 0.489022854 | 0.025086504 | 2.2355165 |
| *Pmch* | 39.820129 | 49.51189 | 14.521915 | 10.832637 | 0.025281521 | 3.523312855 |
| *Atf6* | 1.283407815 | 1.120267237 | 0.701967141 | 0.645551015 | 0.025688275 | 1.783779344 |
| *Dennd2d* | 3.894767887 | 4.180597241 | 2.959433513 | 3.117853995 | 0.025722619 | 1.328777866 |
| *Nt5c* | 4.3889098 | 4.729823595 | 2.353673793 | 1.571068962 | 0.025963732 | 2.323396453 |
| *Cnppd1* | 2.651261722 | 2.666464968 | 2.260023694 | 2.371927437 | 0.026063136 | 1.14805328 |
| *Dnaaf1* | 4.259736 | 4.721674 | 2.136345 | 2.650476 | 0.026093376 | 1.876278641 |
| *Marveld1* | 1.151080244 | 1.435918456 | 0.434638633 | 0.348135898 | 0.026159095 | 3.304909139 |
| *Zbtb24* | 0.604088 | 0.844002 | 0 | 0 | 0.026367789 | #DIV/0! |
| *Prelid1* | 10.720431 | 8.514204 | 2.874575 | 3.028408 | 0.026433126 | 3.258460172 |
| *2810002D19Rik* | 15.085661 | 14.740234 | 6.452727 | 8.843952 | 0.026555436 | 1.949828129 |
| *Adipor1* | 1.960052859 | 1.843384155 | 0.884591283 | 1.154264969 | 0.026624846 | 1.865475808 |
| *Amotl2* | 0.321624471 | 0.368513785 | 0 | 0.089239613 | 0.027009256 | 7.733541544 |
| *Rps6* | 23.02584925 | 27.65849883 | 11.77043731 | 10.56329278 | 0.027349975 | 2.269408105 |
| *Gp1bb* | 0.863084149 | 0.660937314 | 0 | 0.123850287 | 0.027490943 | 12.30535269 |
| *Ndufb10* | 9.401332695 | 9.240062869 | 5.75966225 | 4.294806184 | 0.028213586 | 1.854040886 |
| *Grpel1* | 4.290358 | 4.888497 | 2.483737 | 1.919767 | 0.028380983 | 2.084443434 |
| *Ccnd3* | 2.128918184 | 2.41468291 | 1.460184585 | 1.401141969 | 0.028793803 | 1.587935179 |
| *Mgll* | 0.895512167 | 0.975049173 | 0 | 0.268015656 | 0.029111346 | 6.979298775 |
| *Clip1* | 1.391547896 | 1.763735569 | 0.474220616 | 0.281869702 | 0.029165711 | 4.173156816 |
| *Uqcrb* | 3.845762414 | 3.598252186 | 1.539332523 | 0.652759241 | 0.029370991 | 3.395849902 |
| *Acta2* | 2.820089 | 2.369223 | 0 | 0.656995 | 0.029545683 | 7.898556306 |
| *Rps23* | 249.661682 | 275.472015 | 190.229782 | 183.304871 | 0.029695829 | 1.405850014 |
| *Npc1* | 1.048347936 | 1.286998278 | 0.467249173 | 0.501779199 | 0.0297636 | 2.40998745 |
| *Atp5h* | 5.744776431 | 7.32312242 | 2.161897679 | 1.790714258 | 0.030213782 | 3.306142636 |
| *Plbd1* | 5.55998675 | 4.52717804 | 2.202554188 | 1.930799758 | 0.030701232 | 2.440430924 |
| *Foxo3* | 0.916721164 | 0.719892947 | 0.256351818 | 0.157837762 | 0.030924314 | 3.951364761 |
| *Ppib* | 11.604986 | 13.879923 | 6.218587 | 6.529377 | 0.030993813 | 1.999135627 |
| *Mrpl19* | 4.09463211 | 5.138111927 | 1.029987156 | 1.612184404 | 0.031334858 | 3.494377193 |
| *Klhdc4* | 4.74930759 | 3.791742963 | 1.216237484 | 1.613534672 | 0.031405702 | 3.018282067 |
| *Smap2* | 1.575006424 | 1.254383597 | 0.490858585 | 0.299916429 | 0.031912739 | 3.577996231 |
| *Tbc1d9b* | 0.855996655 | 1.017059404 | 0.339826646 | 0.142307956 | 0.031972777 | 3.884923531 |
| *Lars2* | 99.13958 | 91.214615 | 22.144768 | 43.621075 | 0.032141546 | 2.894423402 |
| *Bora* | 3.605652 | 2.902063 | 0 | 0.782342 | 0.032153598 | 8.318248285 |
| *Podnl1* | 0.854902566 | 1.053743101 | 0.362257491 | 0.220049038 | 0.032332398 | 3.277733583 |
| *Coq4* | 6.319441779 | 5.36067785 | 1.916628694 | 2.776704428 | 0.032344685 | 2.488661965 |
| *Brap* | 0.397717599 | 0.474620466 | 0.223432979 | 0.18489318 | 0.032690713 | 2.136375649 |
| *Fry* | 0.462856821 | 0.492829499 | 0.224113629 | 0.298631853 | 0.032737703 | 1.828205795 |
| *Zfp687* | 2.167600223 | 2.07399683 | 1.453116625 | 1.164090452 | 0.033244756 | 1.620657796 |
| *Nol3* | 3.584639 | 3.494894 | 1.758751 | 0.943028 | 0.033390426 | 2.620322758 |
| *Ybey* | 7.848227 | 8.555894 | 4.527084 | 2.979245 | 0.03469394 | 2.185371971 |
| *Stambpl1* | 1.080496574 | 1.437163756 | 0.310920929 | 0.139582791 | 0.034741949 | 5.588545049 |
| *Gse1* | 4.301497418 | 4.49431982 | 2.801864463 | 3.28590709 | 0.035082329 | 1.444833657 |
| *Dennd4b* | 1.865737957 | 2.247440457 | 1.06744873 | 1.067889741 | 0.035285514 | 1.92624189 |
| *Ngp* | 3600.879395 | 4865.453125 | 147.441315 | 849.735046 | 0.035519215 | 8.490306079 |
| *Srrd* | 17.20997781 | 16.80497564 | 11.43347978 | 13.21613667 | 0.036053846 | 1.379938447 |
| *Foxk2* | 0.94803919 | 0.93585979 | 0.148241683 | 0.407559418 | 0.036149489 | 3.389520056 |
| *Thap8* | 8.482796237 | 10.85925322 | 3.378230803 | 3.734915345 | 0.036513474 | 2.719197533 |
| *Sh3bp4* | 0.915669877 | 1.250646576 | 0.076237262 | 0.226824005 | 0.036725723 | 7.148113877 |
| *Clic3* | 15.50394598 | 13.51617119 | 5.325052215 | 1.375478769 | 0.037076046 | 4.331017531 |
| *Tpm3* | 3.198374727 | 2.597995144 | 1.414098649 | 1.258805772 | 0.037231325 | 2.168566083 |
| *Kctd11* | 0.404736 | 0.49789 | 0 | 0.123698 | 0.037322981 | 7.297013695 |
| *Asns* | 1.886379677 | 2.822641773 | 0 | 0 | 0.037331067 |  |
| *Pcnt* | 1.895629 | 2.483962 | 0.682843 | 0.388534 | 0.037340802 | 4.087815027 |
| *Coro7* | 0.366230526 | 0.427226199 | 0.198536571 | 0.23611036 | 0.037628303 | 1.825520136 |
| *1700037C18Rik* | 45.84590071 | 54.26685122 | 17.82350817 | 25.54889143 | 0.03825152 | 2.308213354 |
| *Ubtf* | 1.595758908 | 1.766262081 | 0.998859628 | 0.710888184 | 0.038661618 | 1.966384144 |
| *Ppp1r2* | 1.462109955 | 0.969554603 | 0 | 0 | 0.038666046 |  |
| *Lbp* | 4.818724948 | 6.152675497 | 1.844969733 | 2.256593179 | 0.038900427 | 2.674931649 |
| *Dnmbp* | 1.210758 | 0.942891 | 0.375982 | 0.19684 | 0.03913134 | 3.759717678 |
| *Psph* | 3.392268836 | 4.430816225 | 1.234705054 | 1.418069985 | 0.039176851 | 2.949019404 |
| *Snapc4* | 0.763017396 | 0.592958116 | 0.254354908 | 0.167815159 | 0.03930863 | 3.211917707 |
| *Hipk1* | 2.511959346 | 3.275053642 | 0.332807409 | 0.877125871 | 0.039471991 | 4.782919092 |
| *Mafg* | 6.092709198 | 5.830359448 | 4.260180774 | 3.434451796 | 0.039512167 | 1.549530603 |
| *Camp* | 276.438873 | 282.582184 | 47.411617 | 126.18354 | 0.039547586 | 3.220257216 |
| *Amfr* | 2.273204196 | 3.112013891 | 0.486377687 | 0.698492829 | 0.039907609 | 4.544984465 |
| *Dars2* | 2.706396178 | 3.421893028 | 0.827689334 | 1.258265345 | 0.04012419 | 2.937882241 |
| *Pmm2* | 1.516826568 | 1.872418883 | 0.797254611 | 0.853381603 | 0.040299514 | 2.053296434 |
| *Hsf2* | 5.447934 | 7.211539 | 2.062951 | 2.090632 | 0.040411527 | 3.047843994 |
| *Snx21* | 9.199306685 | 9.958897386 | 6.265837371 | 4.757681193 | 0.040492417 | 1.737939113 |
| *Apobr* | 15.35852125 | 14.91195854 | 8.917314825 | 11.01811587 | 0.04057525 | 1.518426176 |
| *Dnajc3* | 0.915952 | 0.904387 | 0.439365 | 0.602913 | 0.041650213 | 1.746500454 |
| *Fam20a* | 3.52390025 | 4.64771889 | 1.469315718 | 1.083350299 | 0.041927836 | 3.201209671 |
| *Ide* | 0.403693595 | 0.274483047 | 0.033464097 | 0.03552308 | 0.042172706 | 9.830473853 |
| *Scp2* | 1.67299563 | 2.334534238 | 0.103838229 | 0.431576998 | 0.042359162 | 7.484900811 |
| *Zyg11b* | 0.599039136 | 0.763297691 | 0.257347178 | 0.131107664 | 0.042393417 | 3.50706615 |
| *Tbc1d2b* | 2.261609537 | 1.908412669 | 0.305838806 | 0.845228344 | 0.042708861 | 3.622744517 |
| *Mpp5* | 3.052237 | 3.130422 | 1.476516 | 2.042666 | 0.043089498 | 1.756845483 |
| *Hdac3* | 1.198313353 | 1.309952106 | 0.387209886 | 0.676620162 | 0.043155148 | 2.357768953 |
| *Msra* | 6.624956 | 7.424655 | 1.892521 | 3.574243 | 0.043990903 | 2.570005034 |
| *Pqlc3* | 6.24145495 | 6.821142337 | 3.460852519 | 4.427018553 | 0.044283801 | 1.656035851 |
| *Ikzf1* | 2.145333299 | 1.7762284 | 0.397125591 | 0.850538487 | 0.044632632 | 3.143123033 |
| *Ints7* | 0.795787603 | 0.810192139 | 0.009940788 | 0.294426174 | 0.044705474 | 5.276458827 |
| *Smurf2* | 1.083458447 | 0.875853848 | 0.304849771 | 0.462808038 | 0.044730688 | 2.5523251 |
| *Atxn2l* | 3.637739281 | 3.567436052 | 2.568473019 | 1.993956074 | 0.044771033 | 1.579241055 |
| *Tagln* | 17.258369 | 21.436974 | 9.289358 | 10.063828 | 0.045038558 | 1.99943012 |
| *Zfp597* | 1.088870082 | 1.458340658 | 0.376462432 | 0.116067193 | 0.04514406 | 5.171690404 |
| *Card9* | 5.339402782 | 7.003694659 | 0 | 1.675203153 | 0.045667752 | 7.368119752 |
| *Anxa3* | 3.677766077 | 4.897795426 | 0.727711997 | 1.464544908 | 0.046411143 | 3.911750253 |
| *Tmem115* | 1.478399 | 1.063607 | 0.307354 | 0.361985 | 0.046461488 | 3.797785577 |
| *Calm1* | 4.001897714 | 5.110343351 | 1.019228943 | 1.868552386 | 0.046823804 | 3.155447047 |
| *Sirt1* | 3.696821289 | 3.43923478 | 2.205333941 | 2.652866099 | 0.047738671 | 1.468868307 |
| *Axl* | 1.909413258 | 2.026902057 | 0.872440392 | 1.265825861 | 0.048383774 | 1.8408911 |
| *Pik3cd* | 4.131289179 | 3.695979988 | 0.890412454 | 1.944511864 | 0.048425241 | 2.761015212 |
| *Capn7* | 4.053893172 | 5.310874949 | 1.539920753 | 1.993103007 | 0.048694281 | 2.650638307 |
| *Ppp1r12c* | 1.649593587 | 1.9055354 | 0.380201008 | 0.848125228 | 0.048732582 | 2.894287271 |
| *Tspan32os* | 13.84265804 | 16.33144108 | 6.945333618 | 9.039943266 | 0.04874828 | 1.887618171 |
| *Fbf1* | 0.081696347 | 0.100355143 | 0.040820522 | 0.05020603 | 0.048826617 | 1.999982272 |
| *Ciz1* | 1.592482198 | 2.008770995 | 0.510253378 | 0.822751947 | 0.048837861 | 2.701604506 |
| *Snx12* | 1.877481355 | 1.342316373 | 0.261313141 | 0.464376776 | 0.048843163 | 4.436878138 |
| *Kbtbd11* | 0.947572763 | 1.316824231 | 0.34220166 | 0.325303102 | 0.049625342 | 3.392330841 |
| *Dhx33* | 0.10218542 | 0.072233641 | 0 | 0.019793786 | 0.049907503 | 8.811808878 |

Values are FPKM values. Listed are genes that were found to be present at significantly higher levels in *Cx3cr1*^CreERT2^: *Ppard*^fl/fl^ compared to *Ppard*^fl/fl^ microglia by two-tailed Mann-Whitney U test. (P<0.050).

**Supplementary Table 3: Genes found to be significantly downregulated in *Cx3cr1*^CreERT2^: *Ppard*^fl/fl^ versus *Ppard*^fl/fl^ microglia**

| **gene_name** | **Sample 1**  ***Cx3cr1*^CreERT2^: *Ppard*^fl/fl^** | **Sample 2 *Cx3cr1*^CreERT2^: *Ppard*^fl/fl^** | **Sample 1**  ***Ppard*^fl/fl^** | **Sample 2**  ***Ppard*^fl/fl^** | **P-Value** | **Fold-change** |
| --- | --- | --- | --- | --- | --- | --- |
| *Tcf7l1* | 0 | 0 | 0.292658572 | 0.292794826 | 0.000148161 |  |
| *Creb5* | 0.007081306 | 0 | 0.379868206 | 0.388849408 | 0.000325623 | 0.009211843 |
| *Tradd* | 0 | 0 | 0.289706947 | 0.290032771 | 0.000357791 |  |
| *Zfp518b* | 0 | 0 | 0.113259299 | 0.113093901 | 0.000465184 |  |
| *P2ry1* | 0 | 0 | 0.090824079 | 0.090690238 | 0.000469414 |  |
| *Bdkrb1* | 0 | 0 | 0.267965502 | 0.267498368 | 0.000555382 |  |
| *Cacnb3* | 0 | 0 | 0.245661394 | 0.245225894 | 0.000564788 |  |
| *Snrpa1* | 0 | 0 | 0.651183354 | 0.649999532 | 0.000579199 |  |
| *Sos1* | 0 | 0 | 0.128758 | 0.128521 | 0.000586441 |  |
| *Actbl2* | 0 | 0 | 0.139543 | 0.13928 | 0.000600492 |  |
| *Dlgap1* | 0 | 0 | 0.044954515 | 0.045074403 | 0.000847759 |  |
| *Gng7* | 0 | 0 | 0.092667544 | 0.092998265 | 0.001133994 |  |
| *Anapc2* | 0.522536996 | 0.457768617 | 1.831923461 | 1.907210149 | 0.001453835 | 0.262174534 |
| *5730596B20Rik* | 0 | 0 | 0.113873 | 0.114448 | 0.00160325 |  |
| *Tcf3* | 0 | 0 | 0.285612441 | 0.287081963 | 0.00163355 |  |
| *Frs2* | 0 | 0 | 0.305452 | 0.303773 | 0.001754494 |  |
| *Bco2* | 0 | 0 | 0.209579 | 0.210911 | 0.002016635 |  |
| *Cdc16* | 0 | 0 | 0.44739587 | 0.444488371 | 0.002075342 |  |
| *Fam76b* | 0 | 0 | 0.392026728 | 0.389291709 | 0.00222849 |  |
| *Prox2* | 0 | 0 | 0.355022317 | 0.352444204 | 0.002319926 |  |
| *Trap1* | 0.194761881 | 0.118026957 | 1.299593129 | 1.224365799 | 0.00235785 | 0.123927864 |
| *Usp14* | 0 | 0 | 0.561191329 | 0.565432139 | 0.002396338 |  |
| *Arsa* | 0 | 0 | 0.463859061 | 0.460293046 | 0.002456505 |  |
| *Hbegf* | 0 | 0 | 0.164233 | 0.162875 | 0.002642933 |  |
| *Dclk2* | 0 | 0 | 0.073367914 | 0.074001946 | 0.002738926 |  |
| *Zfp366* | 0 | 0 | 0.282105 | 0.284564 | 0.002762527 |  |
| *Rps19bp1* | 0 | 0 | 2.926752 | 2.900339 | 0.002885646 |  |
| *Bnc2* | 0 | 0 | 0.069610964 | 0.070278587 | 0.003038248 |  |
| *Wdr45* | 0 | 0 | 0.598756224 | 0.604641222 | 0.00311325 |  |
| *Taf3* | 0 | 0 | 0.585189584 | 0.579465559 | 0.003128822 |  |
| *Rrp12* | 0 | 0 | 0.174055 | 0.175821 | 0.003213313 |  |
| *Slco5a1* | 0 | 0 | 0.141094837 | 0.142655374 | 0.003501173 |  |
| *Bicd2* | 0 | 0 | 0.180002 | 0.177989 | 0.003579705 |  |
| *Tmem132a* | 29.68809419 | 26.73723465 | 71.25072003 | 73.06161884 | 0.00380458 | 0.390994487 |
| *Asphd2* | 0 | 0 | 0.984679897 | 0.972438566 | 0.00398186 |  |
| *Yaf2* | 0 | 0 | 0.312114282 | 0.308123684 | 0.004095942 |  |
| *Oacyl* | 0 | 0 | 0.742721211 | 0.752423746 | 0.004131198 |  |
| *Gss* | 0 | 0 | 0.713359205 | 0.704121549 | 0.004148763 |  |
| *9030617O03Rik* | 0 | 0 | 1.113164806 | 1.098682443 | 0.004168293 |  |
| *Aen* | 0 | 0.068201795 | 0.781111222 | 0.72653127 | 0.004468426 | 0.045237379 |
| *Kdm4d* | 0 | 0 | 0.201606912 | 0.204494075 | 0.004525954 |  |
| *Exoc2* | 0 | 0 | 0.265276265 | 0.261494946 | 0.004569766 |  |
| *Nat9* | 1.110140987 | 1.183659626 | 3.860443949 | 3.712708762 | 0.0046037 | 0.302885826 |
| *Rpl3l* | 0 | 0 | 0.644475806 | 0.653880706 | 0.004611399 |  |
| *Fgfr1* | 0 | 0 | 0.095991299 | 0.094546961 | 0.004825679 |  |
| *Slc39a6* | 0 | 0 | 0.186267376 | 0.189139084 | 0.004869789 |  |
| *Gcnt1* | 0 | 0 | 0.138513193 | 0.140657038 | 0.004888727 |  |
| *Ccdc12* | 0.221923795 | 0.106792245 | 1.33863185 | 1.234597841 | 0.004944071 | 0.127744539 |
| *Mapkapk5* | 0 | 0 | 0.153861603 | 0.151464999 | 0.004996925 |  |
| *Azin1* | 1.016626839 | 0.938836496 | 2.207057137 | 2.09647246 | 0.005222534 | 0.454385938 |
| *Mocs2* | 0 | 0 | 0.125977773 | 0.128067963 | 0.005237743 |  |
| *Inca1* | 0 | 0 | 1.507592989 | 1.533356741 | 0.005393486 |  |
| *Spint1* | 0 | 0 | 0.813165067 | 0.827480808 | 0.005554796 |  |
| *Mettl17* | 0 | 0 | 0.192409284 | 0.189069478 | 0.005573396 |  |
| *E130309D02Rik* | 0 | 0 | 0.164661963 | 0.167600033 | 0.005629247 |  |
| *Naaladl1* | 0 | 0 | 0.194305 | 0.190802 | 0.005790644 |  |
| *Fgf6* | 0 | 0 | 0.082012 | 0.083562 | 0.005959461 |  |
| *Prr5* | 0 | 0 | 0.200588811 | 0.204381502 | 0.005961997 |  |
| *Hcar1* | 0 | 0 | 0.234114 | 0.238541 | 0.005962559 |  |
| *Aaed1* | 0 | 0 | 0.400953 | 0.408535 | 0.00596267 |  |
| *Mfge8* | 0 | 0 | 0.081365844 | 0.08290448 | 0.005962718 |  |
| *Gprc5d* | 0 | 0 | 0.275761869 | 0.280976731 | 0.005962919 |  |
| *Chpf2* | 0 | 0 | 0.033942644 | 0.034584703 | 0.005964564 |  |
| *Prrx1* | 0 | 0 | 0.034514181 | 0.035167117 | 0.005965155 |  |
| *Fat2* | 0 | 0 | 0.0516 | 0.052583 | 0.006006533 |  |
| *Dnaaf3* | 0 | 0 | 0.528503 | 0.518501 | 0.006081426 |  |
| *Sepsecs* | 0 | 0 | 0.047910396 | 0.048847662 | 0.006166551 |  |
| *Grip2* | 0 | 0 | 0.367404689 | 0.360072877 | 0.006415893 |  |
| *Treml4* | 0 | 0 | 0.187026959 | 0.190892515 | 0.006511448 |  |
| *Ankrd55* | 0 | 0 | 0.412253752 | 0.403680737 | 0.006688711 |  |
| *Lzic* | 0 | 0 | 1.016734274 | 1.038493399 | 0.006739775 |  |
| *Slc39a1* | 0 | 0 | 1.691453 | 1.652887 | 0.007340997 |  |
| *Brat1* | 0 | 0 | 0.123201553 | 0.12038651 | 0.007356814 |  |
| *Spata17* | 0 | 0 | 0.121676259 | 0.124525558 | 0.007367285 |  |
| *Arf6* | 0.81525 | 0.835585 | 1.623833 | 1.625998 | 0.00740906 | 0.507975646 |
| *Fads6* | 0 | 0 | 0.2269155 | 0.232379981 | 0.007573845 |  |
| *Tspo2* | 0 | 0 | 0.473660215 | 0.485116964 | 0.007606821 |  |
| *Itga2b* | 0.020047276 | 0 | 0.263294114 | 0.293554665 | 0.007889317 | 0.036001293 |
| *Prr29* | 0 | 0 | 0.329479634 | 0.3378227 | 0.007959037 |  |
| *Zscan22* | 0 | 0 | 0.790990536 | 0.77128051 | 0.008031338 |  |
| *Ipo4* | 0.124043582 | 0 | 1.066018559 | 0.94960823 | 0.008104921 | 0.061540947 |
| *Fndc3b* | 0.213458075 | 0.197151246 | 0.482493421 | 0.511021542 | 0.008107893 | 0.413289519 |
| *Olfr1123* | 0 | 0 | 0.392935 | 0.403087 | 0.008118637 |  |
| *Gm6578* | 0 | 0 | 0.388142602 | 0.39817252 | 0.008120025 |  |
| *Ccr10* | 0 | 0 | 0.883622 | 0.906464 | 0.008123006 |  |
| *Grap2* | 0 | 0 | 0.120119 | 0.123227 | 0.008130426 |  |
| *Spcs1* | 0 | 0 | 0.490664589 | 0.503433404 | 0.008176692 |  |
| *Nme2* | 0 | 0 | 2.802114787 | 2.729840184 | 0.008316919 |  |
| *Pi16* | 0 | 0 | 0.293936006 | 0.286228503 | 0.008457016 |  |
| *Fam131b* | 0 | 0 | 0.186126 | 0.181115 | 0.008686132 |  |
| *Ttl* | 0 | 0 | 0.191857788 | 0.197267513 | 0.00884989 |  |
| *Fez2* | 0 | 0 | 0.46227 | 0.449586 | 0.008854868 |  |
| *Nup43* | 0 | 0 | 1.084913806 | 1.115587379 | 0.008873496 |  |
| *Ppard* | 0 | 0 | 0.084156502 | 0.081745835 | 0.00924984 |  |
| *Dgkz* | 0 | 0.112162189 | 0.919099721 | 0.808980548 | 0.009340272 | 0.064905659 |
| *Slit3* | 0 | 0 | 0.062952286 | 0.064838575 | 0.0093963 |  |
| *Nup188* | 0 | 0 | 0.239724079 | 0.246994783 | 0.009509246 |  |
| *Snrpb* | 0 | 0 | 0.763938987 | 0.787761371 | 0.009772898 |  |
| *Dnajb12* | 0 | 0 | 0.889508479 | 0.862226221 | 0.009914184 |  |
| *Map7* | 0 | 0 | 0.085391455 | 0.082748619 | 0.010005607 |  |
| *Mical3* | 0 | 0 | 0.016998865 | 0.017554276 | 0.010232216 |  |
| *Pcdh19* | 0 | 0 | 0.036499205 | 0.037694869 | 0.010258464 |  |
| *Kcnk12* | 0 | 0 | 0.19728 | 0.203745 | 0.010262179 |  |
| *Rhbdl1* | 0 | 0 | 0.440316863 | 0.454788368 | 0.010291576 |  |
| *Ubl7* | 0 | 0 | 0.276199 | 0.285303 | 0.010321029 |  |
| *Phospho1* | 0 | 0 | 0.502911755 | 0.486665929 | 0.010450402 |  |
| *Cr2* | 0 | 0 | 0.187907195 | 0.19427554 | 0.01060707 |  |
| *Rpap2* | 0 | 0 | 0.648094979 | 0.626439093 | 0.010815904 |  |
| *Ikbip* | 0 | 0 | 0.626450622 | 0.604936768 | 0.0111214 |  |
| *Uqcrc1* | 0 | 0 | 1.126419099 | 1.167119504 | 0.011296066 |  |
| *Sall3* | 0 | 0 | 0.189379 | 0.196454 | 0.011672356 |  |
| *Gm4876* | 10.89460881 | 9.312325679 | 20.67376851 | 19.35442487 | 0.011778817 | 0.504817549 |
| *Gm7008* | 112.1170816 | 108.3572831 | 173.7187635 | 172.674824 | 0.012016268 | 0.636485122 |
| *Bmp7* | 0 | 0 | 0.093130968 | 0.096827183 | 0.012385817 |  |
| *Mapre3* | 0 | 0 | 0.110418076 | 0.114800827 | 0.012387032 |  |
| *Aplnr* | 0 | 0 | 0.101144 | 0.105204 | 0.012524196 |  |
| *Mrps17* | 0 | 0.003830452 | 0.060049662 | 0.061185073 | 0.012541071 | 0.031595333 |
| *Stx19* | 0 | 0 | 0.865752 | 0.832225 | 0.012568591 |  |
| *Siglec1* | 0 | 0 | 0.052014304 | 0.05411022 | 0.012571346 |  |
| *Anxa7* | 0 | 0 | 1.513026 | 1.574601 | 0.012694107 |  |
| *Atg14* | 0 | 0 | 0.572353 | 0.549866 | 0.012754866 |  |
| *Tpd52* | 0.090358328 | 0.104607225 | 0.505462066 | 0.470131275 | 0.013061426 | 0.199843054 |
| *Isl1* | 0 | 0 | 0.086176972 | 0.08979114 | 0.013073548 |  |
| *Tnni3* | 0 | 0 | 0.188912814 | 0.180935475 | 0.013729264 |  |
| *Gxylt2* | 0 | 0 | 0.043658699 | 0.045730228 | 0.014750604 |  |
| *Hepacam2* | 0 | 0 | 0.062809959 | 0.065799314 | 0.014794731 |  |
| *Camk2n1* | 0 | 0 | 0.123362 | 0.129268 | 0.014880227 |  |
| *Nlrp5-ps* | 0 | 0 | 0.039557398 | 0.041526062 | 0.015453762 |  |
| *Grb7* | 0 | 0 | 0.425880297 | 0.405675307 | 0.015465431 |  |
| *Lacc1* | 1.377689641 | 1.087311187 | 6.591023433 | 6.552645456 | 0.015507158 | 0.187542828 |
| *Cirbp* | 0 | 0 | 1.37499501 | 1.309013144 | 0.015647084 |  |
| *Colgalt2* | 0 | 0 | 0.369125927 | 0.350891106 | 0.016119295 |  |
| *Tars2* | 0 | 0 | 0.244749965 | 0.232301163 | 0.016609027 |  |
| *Tanc1* | 0 | 0 | 0.106102023 | 0.111981956 | 0.017160246 |  |
| *Tifab* | 0.280998 | 0.155576 | 0.994336 | 0.855948 | 0.017469505 | 0.235949724 |
| *Snx13* | 0 | 0 | 0.203002927 | 0.191965072 | 0.017786474 |  |
| *Cnih1* | 0 | 0 | 1.231238 | 1.161497 | 0.018550292 |  |
| *Egf* | 0 | 0 | 0.128692801 | 0.12116066 | 0.019185879 |  |
| *Elk1* | 0 | 0 | 0.068081247 | 0.072320253 | 0.019215005 |  |
| *Nxpe5* | 0 | 0 | 0.239042478 | 0.253948674 | 0.019243122 |  |
| *E230016K23Rik* | 0 | 0 | 0.686844099 | 0.730296776 | 0.019514058 |  |
| *Trabd* | 0 | 0 | 0.270458837 | 0.287844294 | 0.019817818 |  |
| *Sis* | 0 | 0 | 0.102339831 | 0.096148447 | 0.019851449 |  |
| *Trpm7* | 0 | 0 | 0.543287584 | 0.510315868 | 0.01991603 |  |
| *Ppp1r12a* | 1.876871 | 2.346847 | 4.674661 | 4.199279 | 0.020052379 | 0.475968735 |
| *9930012K11Rik* | 0 | 0 | 0.772438124 | 0.725176236 | 0.020083856 |  |
| *Tsen34* | 0 | 0 | 3.004247868 | 3.201569859 | 0.020235331 |  |
| *Il7* | 0 | 0 | 0.384139302 | 0.360067609 | 0.020584565 |  |
| *Slc7a5* | 0.092144831 | 0 | 0.618713144 | 0.569014413 | 0.020648807 | 0.07758078 |
| *Ube2q1* | 0.320886351 | 0.25792123 | 0.609609767 | 0.554922123 | 0.020712828 | 0.497030254 |
| *Naa20* | 3.276099714 | 3.580334148 | 4.72930511 | 5.028542306 | 0.02096927 | 0.702658442 |
| *Zmynd15* | 15.92140264 | 19.53126926 | 36.81720317 | 34.38562828 | 0.02110253 | 0.497910984 |
| *Gm9889* | 0 | 0 | 0.120119 | 0.11233 | 0.021324148 |  |
| *Etl4* | 0.021265949 | 0 | 0.11659233 | 0.101331147 | 0.022476624 | 0.097584477 |
| *Nudt6* | 0 | 0 | 1.289117195 | 1.385168382 | 0.022855379 |  |
| *Inpp5k* | 0.655124589 | 0.547207588 | 1.108363614 | 1.174519945 | 0.022978999 | 0.526672581 |
| *Tcf7l2* | 0 | 0 | 0.523964229 | 0.56340131 | 0.023079107 |  |
| *Rtn2* | 0 | 0 | 0.435457895 | 0.468374189 | 0.023174556 |  |
| *Nmi* | 0 | 0 | 0.953055436 | 1.025306264 | 0.023239367 |  |
| *Zbed5* | 0 | 0 | 0.083107399 | 0.077141823 | 0.023688417 |  |
| *BC005624* | 0 | 0 | 0.874508758 | 0.943955007 | 0.024300391 |  |
| *Adssl1* | 0.642340548 | 0 | 3.363252348 | 2.888172642 | 0.024379288 | 0.102751061 |
| *Fbxo9* | 0 | 0 | 0.521605145 | 0.483037264 | 0.024427621 |  |
| *Itsn2* | 0 | 0 | 0.610595 | 0.565366 | 0.024473167 |  |
| *Angptl3* | 0 | 0 | 1.643094533 | 1.519612374 | 0.024843047 |  |
| *Ldlrad4* | 0 | 0 | 0.306403 | 0.33131 | 0.024851671 |  |
| *Chad* | 0 | 0 | 2.365119 | 2.183399 | 0.02542038 |  |
| *Pcna* | 0.41759927 | 0 | 2.302634992 | 2.52210887 | 0.025561855 | 0.086553666 |
| *Cnn3* | 0.112503568 | 0 | 0.497050655 | 0.61703709 | 0.026180942 | 0.100982682 |
| *Cdca7* | 0 | 0 | 2.013009137 | 1.849475523 | 0.026937736 |  |
| *Nbeal2* | 0.019312052 | 0 | 0.334284599 | 0.286716677 | 0.026952519 | 0.031098249 |
| *Hnf1a* | 0 | 0 | 0.304119675 | 0.279388791 | 0.026965766 |  |
| *Agbl3* | 0 | 0 | 0.186678798 | 0.171340485 | 0.027257488 |  |
| *Fam43a* | 0 | 0 | 0.866059 | 0.794623 | 0.027368004 |  |
| *Papss1* | 0 | 0 | 0.246797145 | 0.269144273 | 0.027556882 |  |
| *Ssu72* | 0 | 0 | 1.457114847 | 1.590066005 | 0.027758671 |  |
| *Serpine1* | 0.08815021 | 0 | 0.450459117 | 0.392723425 | 0.02776155 | 0.104544634 |
| *Sytl2* | 0 | 0 | 0.013045559 | 0.014241641 | 0.027887154 |  |
| *Rce1* | 0 | 0 | 0.229991 | 0.251182 | 0.028018816 |  |
| *Pdik1l* | 0 | 0 | 0.133709888 | 0.146258045 | 0.0285142 |  |
| *Cdon* | 0.044285755 | 0 | 0.216068046 | 0.176372917 | 0.028735674 | 0.112846923 |
| *Ikbke* | 0 | 0 | 0.158460835 | 0.173492654 | 0.028808315 |  |
| *Prdx4* | 0 | 0 | 1.568466038 | 1.719501762 | 0.029223152 |  |
| *Pcolce* | 0.218851708 | 0.199286457 | 0.907632039 | 0.994248534 | 0.029229848 | 0.219855111 |
| *Mrpl4* | 0 | 0 | 2.49434 | 2.271766 | 0.029708139 |  |
| *Imp4* | 0.03994859 | 0 | 0.158265092 | 0.198454023 | 0.030544455 | 0.111988925 |
| *Ffar2* | 0 | 0 | 1.075590491 | 1.1841713 | 0.030565861 |  |
| *B4galnt2* | 0 | 0 | 0.772514 | 0.850846 | 0.03069501 |  |
| *Tmem104* | 0 | 0 | 0.901181105 | 0.99267792 | 0.030732713 |  |
| *Nars* | 0 | 0 | 1.355358 | 1.494642 | 0.031087879 |  |
| *Il17re* | 0 | 0 | 0.064201402 | 0.070828269 | 0.031218416 |  |
| *Iqcb1* | 0.137221744 | 0.433182454 | 1.535941371 | 1.260976074 | 0.031753908 | 0.203940306 |
| *Ifit2* | 0.578878607 | 0 | 2.586267699 | 3.303905862 | 0.031846554 | 0.098278701 |
| *Cox17* | 0 | 0 | 0.296136967 | 0.267803323 | 0.031958354 |  |
| *Dnajc16* | 0 | 0 | 0.237068325 | 0.262163798 | 0.031974781 |  |
| *Rnpc3* | 0 | 0 | 0.343112627 | 0.310180182 | 0.032064816 |  |
| *1700017D01Rik* | 0 | 0 | 0.520468612 | 0.57582074 | 0.032115938 |  |
| *Ogg1* | 0.735925622 | 0 | 4.231188058 | 3.307457184 | 0.032205301 | 0.097620408 |
| *Fan1* | 0 | 0 | 0.139111741 | 0.153954232 | 0.032214442 |  |
| *Tcirg1* | 0.368934253 | 0 | 1.693285739 | 1.377510968 | 0.032468078 | 0.120142845 |
| *Gm9949* | 0 | 0 | 1.13147 | 1.02012 | 0.03291725 |  |
| *Kmo* | 0 | 0 | 0.410296252 | 0.369299507 | 0.033447232 |  |
| *Atg101* | 0 | 0 | 1.404705 | 1.263389 | 0.033687185 |  |
| *Mthfsd* | 0 | 0 | 0.215246723 | 0.193559093 | 0.033741797 |  |
| *Dap* | 0.530109597 | 0.813696361 | 3.272336345 | 3.290490668 | 0.033866174 | 0.204760228 |
| *Rasa2* | 0 | 0 | 0.120778912 | 0.134402876 | 0.033956413 |  |
| *Nhlrc3* | 0.134462549 | 0 | 0.607754143 | 0.488724517 | 0.034180886 | 0.12263125 |
| *Denr* | 0 | 0 | 0.315072041 | 0.351439123 | 0.034701698 |  |
| *Bop1* | 0 | 0 | 3.837941 | 3.437184 | 0.035033383 |  |
| *Slc35e4* | 0 | 0 | 0.679995 | 0.759708 | 0.035212201 |  |
| *3110040N11Rik* | 0 | 0 | 0.971216723 | 1.086728478 | 0.035695793 |  |
| *Spc25* | 0 | 0 | 0.385343815 | 0.431812365 | 0.036163187 |  |
| *Leap2* | 0 | 0 | 3.302015 | 3.702417 | 0.03635223 |  |
| *Zfp276* | 1.671732174 | 2.227083754 | 4.130262228 | 3.756487948 | 0.036553529 | 0.494350124 |
| *Dhrs7b* | 0 | 0 | 0.919716414 | 0.819271682 | 0.036730632 |  |
| *Dnajc8* | 0 | 0 | 0.500297814 | 0.445533973 | 0.036819299 |  |
| *Lipm* | 0 | 0 | 0.45788 | 0.4075 | 0.037020434 |  |
| *Srp14* | 0 | 0 | 0.595009946 | 0.529459895 | 0.037069278 |  |
| *Tmem190* | 0 | 0 | 0.656868894 | 0.583561791 | 0.037579313 |  |
| *Snx9* | 0 | 0 | 1.509973 | 1.341283 | 0.037620737 |  |
| *Ppid* | 0 | 0 | 0.5355457 | 0.603321595 | 0.037841674 |  |
| *Znrd1as* | 0 | 0 | 0.573127294 | 0.645984341 | 0.038000733 |  |
| *Ppm1n* | 0 | 0 | 0.85112487 | 0.959486062 | 0.03805494 |  |
| *Tmem198b* | 4.556981527 | 5.05051874 | 9.363842273 | 10.49383091 | 0.038915102 | 0.483818027 |
| *Alg9* | 0 | 0 | 0.932782385 | 0.82517155 | 0.038921275 |  |
| *Gale* | 0 | 0.162980367 | 2.763805241 | 2.29879881 | 0.038980173 | 0.032192991 |
| *Cd2bp2* | 0 | 0 | 0.288829564 | 0.326561518 | 0.038984761 |  |
| *Ltbp2* | 0 | 0 | 0.125449817 | 0.142004624 | 0.03935507 |  |
| *Dck* | 0.034941256 | 0 | 0.206150291 | 0.217049879 | 0.039807533 | 0.082564372 |
| *Cox8a* | 0 | 0 | 12.070805 | 13.688266 | 0.039922151 |  |
| *Pcnx* | 0 | 0 | 0.38135 | 0.336165 | 0.040037809 |  |
| *Cd9* | 0 | 0 | 0.757827397 | 0.859986303 | 0.040146862 |  |
| *Tulp2* | 1.167525684 | 2.448525179 | 7.643598314 | 6.080959404 | 0.040698644 | 0.263473034 |
| *Fchsd1* | 0 | 0 | 0.806380623 | 0.707655985 | 0.041452899 |  |
| *Cul4b* | 0.12527092 | 0 | 0.545732511 | 0.473984003 | 0.042216166 | 0.12284877 |
| *Saysd1* | 0 | 0 | 0.282339 | 0.323437 | 0.043124469 |  |
| *Mmrn2* | 0 | 0 | 0.726963 | 0.832874 | 0.04315945 |  |
| *Ecd* | 0.215755 | 0 | 1.580524 | 1.551956 | 0.043597453 | 0.068876737 |
| *Tbc1d25* | 0.239683805 | 0 | 1.242121452 | 1.149268962 | 0.04393606 | 0.100227802 |
| *Prkag3* | 0 | 0 | 0.117502618 | 0.102301193 | 0.043958032 |  |
| *Nab2* | 1.561374292 | 0.536218452 | 4.854831431 | 4.251406505 | 0.044092755 | 0.230346797 |
| *Abcb7* | 0 | 0 | 0.058519333 | 0.06724776 | 0.044111649 |  |
| *Dph2* | 0 | 0 | 0.250385725 | 0.287795261 | 0.044181056 |  |
| *Mgrn1* | 0 | 0 | 0.463933 | 0.403592 | 0.044209105 |  |
| *Polr1d* | 0 | 0 | 1.813083267 | 1.576805761 | 0.04430117 |  |
| *2610001J05Rik* | 0.134236814 | 0.20978757 | 0.707907738 | 0.866810488 | 0.044602225 | 0.218467265 |
| *Sertad2* | 0 | 0 | 0.39430774 | 0.341749292 | 0.045381066 |  |
| *Dmtf1* | 0.554529427 | 0.871946385 | 2.581405116 | 2.642312381 | 0.045807163 | 0.273076753 |
| *Pign* | 0.004188741 | 0 | 0.027329329 | 0.028040532 | 0.046232462 | 0.075650203 |
| *Trim3* | 0 | 0 | 0.594242958 | 0.513352929 | 0.046411267 |  |
| *Slc35d1* | 0 | 0 | 0.261777859 | 0.225989466 | 0.046626423 |  |
| *Naa60* | 0 | 0 | 0.746050664 | 0.643263739 | 0.047013972 |  |
| *Uhrf1bp1* | 0.57822723 | 0.797820106 | 1.443786807 | 1.301029997 | 0.047399926 | 0.501325747 |
| *Dcaf8* | 0.686305258 | 0.61004364 | 1.080441696 | 1.095164109 | 0.047565944 | 0.595856517 |
| *Taf4a* | 0 | 0 | 0.199038227 | 0.171292254 | 0.047608006 |  |
| *Atat1* | 0 | 0 | 0.592410618 | 0.509753842 | 0.047654066 |  |
| *Xrcc4* | 0.038927676 | 0.184631236 | 0.88777954 | 0.670179978 | 0.047804023 | 0.143494686 |
| *Trmt6* | 0 | 0 | 0.310118464 | 0.361398846 | 0.048521261 |  |
| *Rbfa* | 0 | 0 | 1.484942 | 1.270783 | 0.049375164 |  |
| *Lrp4* | 0 | 0 | 0.107845363 | 0.092242371 | 0.049543826 |  |
|  |  |  |  |  |  |  |

Values are FPKM values. Listed are genes that were found to be present at significantly lowered levels in *Cx3cr1*^CreERT2^: *Ppard*^fl/fl^ compared to *Ppard*^fl/fl^ microglia by two-tailed Mann-Whitney U test (P<0.050).

**Supplementary Table 4: Significantly Upregulated Biological Processes in *Cx3cr1*^CreERT2^: *Ppard*^fl/fl^ versus *Ppard*^fl/fl^ microglia**

| **GO BP** | **Term description** | **Genes** | **Count** | **P-Value** |
| --- | --- | --- | --- | --- |
| GO:0001934 | positive regulation of protein phosphorylation | Camp,C3, Ccnd3, Fam20a, Hdac3, Mmp9, Spn, Sirt1, Akt2 | 9 | 0.00088 |
| GO:0032820 | negative regulation of tumor necrosis factor production | Axl, Hdac3, Lbp, Sirt1, Tspo | 5 | 0.0012 |
| GO:0043066 | negative regulation of apoptotic process | Axl, Braf, Cbl, Dnajc3, Prelid1, Apc, Asns | 15 | 0.0034 |
|  |  | Cxcr2, Flna, Mmp9, No13, Pcnt, Rps6, Sirt1, Akt2 |  |  |
| GO:0051259 | protein oligomerization | Amfr, Chp1, Dnm1l, Mmp9, Nol3 | 5 | 0.0059 |
| GO:0006869 | lipid transport | Prelid1, Apobr, Lbp, Mttp, Scp2, Tspo | 6 | 0.0071 |
| GO:0045766 | positive regulation of angiogenesis | Anxa3, Camp, Cxcr2, C3, Mmp9, Sirt1 | 6 | 0.01 |
| GO:0031116 | positive regulation of microtubule polymerization | Clip1, Apc, Mapt | 3 | 0.018 |
| GO:0043065 | positive regulation of apoptotic process | Atf6, Apc, Dnm1l, Foxo3, Mmp9, Rps6, Sirt1, Scp2, Tspo | 9 | 0.029 |
| GO:0007052 | mitotic spindle organization | Kif2a, Pcnt, Tubg1 | 3 | 0.036 |
| GO:0042127 | regulation of cell proliferation | Bricd5, Braf, Ccnd3, Hp1bp3, Kctd11, Sirt1, Mafg | 7 | 0.037 |
| GO:0043388 | positive regulation of DNA binding | Calm1, Hipk1, Mmp9 | 3 | 0.046 |

Genes present at significantly higher levels in *Cx3cr1*^CreERT2^: *Ppard*^fl/fl^ compared to *Ppard*^fl/fl^ microglia by two-tailed Mann-Whitney test were entered into DAVID and the list of GO: Biological Processes (BP) that were found to be significant are presented (uncorrected values).

**Supplementary Table 5: Significantly Downregulated Biological Processes in *Cx3cr1*^CreERT2^: *Ppard*^fl/fl^ versus *Ppard*^fl/fl^ microglia**

| **GO term** | **Term description** | **Genes** | **Count** | **P-Value** |
| --- | --- | --- | --- | --- |
| GO:0098779 | mitophagy in response to mitochondrial depolarization | Cd93, Nme2, Atg14, Cox8a, Fam131b, Hcar1, Snrpb | 7 | 0.0058 |
| GO:0030326 | embryonic limb morphogenesis | Hfn1a, Bmp7, Fgfr1, Lrp4, Prrx1 | 5 | 0.0075 |
| GO:0048762 | mesenchymal cell differentiation | Isl1, Bmp7, Fgfr1 | 3 | 0.0077 |
| GO:0006914 | autophagy | Tbc1d25, Wdr45, Anxa7, Arsa, Atg101, Atg14, Dap | 7 | 0.0082 |
| GO:0008543 | fibroblast growth factor receptor signaling pathway | Fgf5, Fgfr1, Frs2, Sos1 | 4 | 0.016 |
| GO:0008283 | cell proliferation | Brat1, Naa60, Anxa7, Bop1, Ecd, Map7, Ppard, Tcf7l2 | 8 | 0.019 |
| GO:0001525 | angiogenesis | Angptl3, Egf, Fgf6, Fgfr1, Hbegf, Mfge8, Mmrn2, Serpine1 | 8 | 0.029 |
| GO:0010212 | response to ionizing radiation | Brat1, Xrcc4, Aen, Lzic | 4 | 0.033 |
| GO:0098792 | xenophagy | Cd93, Cox8a, Fam131b, Hcar1, Snrpb | 5 | 0.038 |
| GO:0072659 | protein localization to plasma membrane | Cacnb3, Map7, P2ry1, Sytl2 | 4 | 0.041 |
| GO:0048146 | positive regulation of fibroblast proliferation | Xrcc4, Egf, Fndc3b, Serpine1 | 4 | 0.046 |
| GO:0045682 | regulation of epidermis development | Nme2, Nab2 | 2 | 0.048 |
|  |  |  |  |  |

Biological processes (BP) found to be present at significantly lower levels in *Cx3cr1*^CreERT2^: *Ppard*^fl/fl^ compared to *Ppard*^fl/fl^ by two-tailed Mann-Whitney test were entered into DAVID and the list of GO: Biological Processes that were found to be significant are presented (uncorrected values).
